# Interannual variability of net primary productivity in the northwest African coastal upwelling system and their relation to Dakar Niños

**DOI:** 10.1038/s41598-025-31860-y

**Published:** 2025-12-16

**Authors:** Rodrigue Anicet Imbol Koungue, Arthur Prigent, Joke F. Lübbecke, Peter Brandt, Julien Jouanno

**Affiliations:** 1 GEOMAR Helmholtz Centre for Ocean Research Kiel, Kiel, Germany; 2 Earth System Physics, The Abdus Salam International Centre for Theoretical Physics, Trieste, Italy; 3 Institute of Environmental Physics, University of Bremen, Bremen, Germany; 4 MARUM – Center for Marine Environmental Sciences, University of Bremen, Bremen, Germany; 5 Faculty of Mathematics and Natural Sciences, Kiel University, Kiel, Germany; 6 LEGOS, IRD, CNRS, CNES, Université de Toulouse, UPS, Toulouse, France

**Keywords:** Climate sciences, Ecology, Ecology, Ocean sciences

## Abstract

The Canary Current upwelling system, located along the northwest African coast between approximately 10ºN and 35ºN, is among the most productive marine ecosystems globally, supporting a rich marine biodiversity. In the southern part (9ºN–18ºN), pronounced interannual variability in net primary production (NPP) is influenced by extreme warm and cold events, known as Dakar Niños and Niñas, respectively. In this study, we analyze the physical mechanisms driving the interannual variability of NPP from 2003 to 2023, using a combination of satellite observations, reanalysis data, and ocean model outputs. Our results indicate that the interannual NPP variability is closely linked to changes in sea surface temperature, with the most pronounced effects occurring during March-April-May, i.e., the main upwelling season. A total of six undocumented extreme coastal low NPP events are identified, nearly all of which are associated with Dakar Niños. These low NPP events are linked to both local and remote forcing. The local forcing is associated with fluctuations of alongshore winds and near-coastal wind stress curl. The remote forcing involves the propagations of coastal trapped waves emanating from the equator and the northern Gulf of Guinea. Part of the local and remote forcing can be associated with large-scale climate modes.

**Supplementary Information:**

The online version contains supplementary material available at 10.1038/s41598-025-31860-y.

## Introduction

**Fig. 1:**
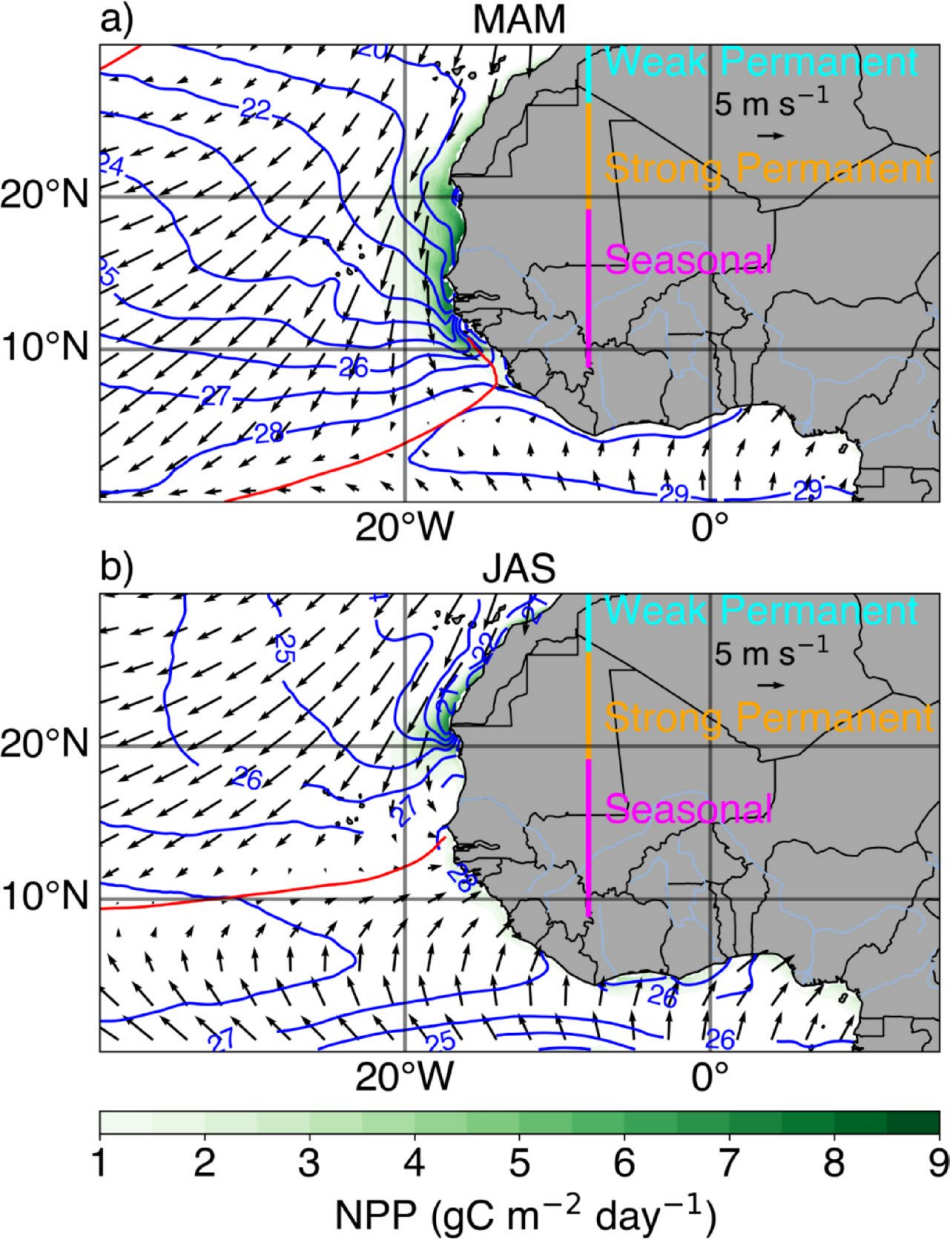
(**a**) Climatological mean NPP (shading), Optimum Interpolation sea surface temperature (OI-SST, blue contours, every 1 °C), and ERA5 wind field at 10 m (arrows) during the upwelling season from March to May. The position of the ITCZ is denoted by the red contour which indicates the 0 m s^−1^ meridional wind speed at 10 m. (**b**) Same as (a) but during the relaxation season from July to August. The climatological means are calculated over the period 2003-2023.

The Canary Current upwelling system (CCUS), located along the northwestern coast of Africa, is among the most productive marine ecosystems globally, supporting local and regional fisheries^1,2^. Based on the upwelling intensity throughout the year, the CCUS can be divided into three subregions (Fig. 1; adapted from *Gómez-Letona *et al.^3^): a weak permanent upwelling zone (from 26°N to 33°N), a permanent upwelling zone (18°N to 26°N), and a seasonal upwelling zone (from 9°N to 18°N), the latter being the focus of the present study.

**Fig. 2:**
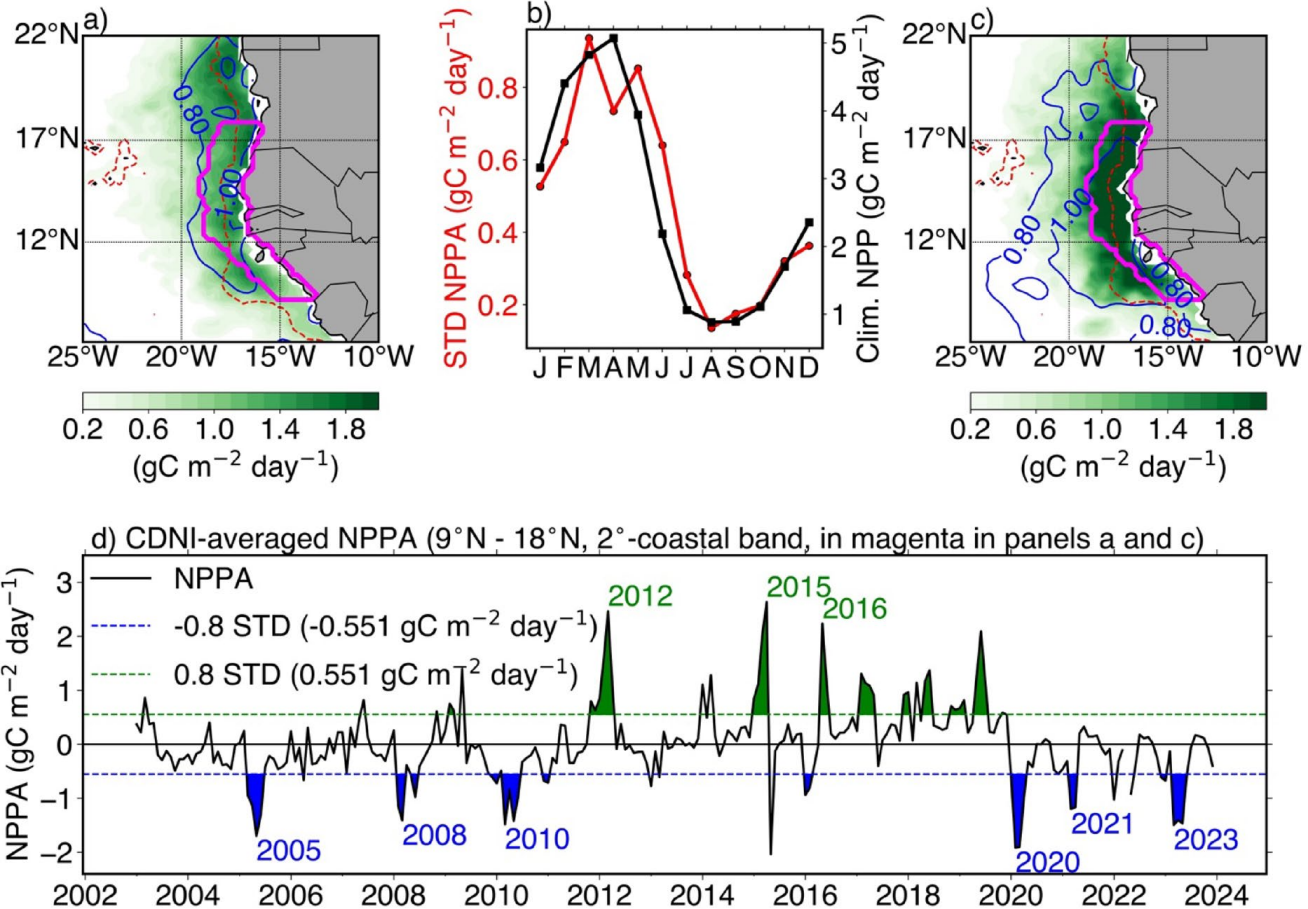
(**a**) Standard deviation of detrended net primary production anomalies (NPPA, shading) estimated by the standard vertically generalized production model and OI-SST anomalies (SSTA, blue contours, every 0.2 °C). The magenta region in (a) represents the coastal Dakar Niño Index region (CDNI, 9°N-18°N, 2°-coastal band). The dashed red line represents the isobath 2000 m. (**b**) Seasonal cycle of the standard deviation of CDNI-averaged detrended NPPA (red) and climatology (black) of the CDNI-averaged detrended NPP. (**c**) Same as (a), but for NPPA averaged in March-April-May (MAM) and SSTA averaged in February-March-April (FMA). (**d**) Monthly detrended CDNI-averaged NPPA. The horizontal green and blue dashed lines indicate the ± 0.8 standard deviation of the interannual NPPA. Green and blue shaded areas represent the extreme high and low NPP events, respectively, and are identified when the CDNI-averaged NPPA exceeds ± 0.8 standard deviation (± 0.551 gC m^−2^ day^−1^) for at least 2 consecutive months. Only the NPPA events that peak during MAM are highlighted by their corresponding years. All anomalies have been calculated over the period 2003/01–2023/12.

The pronounced seasonality in the southern part of the CCUS depends mostly on the strength and position of the Azores high-pressure system^4^, and the seasonal migration of the Intertropical Convergence Zone (ITCZ)^5^. From March to May (MAM), the ITCZ is closest to the equator, allowing for strong upwelling favourable alongshore winds resulting in high net primary production (NPP; Fig. 1a). After its northward migration, the ITCZ reaches its northernmost position at about 12°N from July to September, leading to weak alongshore winds, i.e., unfavourable for upwelling, and to low NPP along the coasts of Senegal and Mauritania (Fig. 1b). The seasonal upwelling zone features large interannual sea surface temperature (SST) variability dominated by extreme coastal warm and cold events called Dakar Niños and Niñas^6^, respectively. Dakar Niños/Niñas typically peak in February-March-April during the early upwelling season in the coastal Dakar Niño index (CDNI, 2°-width coastal band from 9°N and 18°N, magenta domain in Fig. 2a) region. These events are reported to be driven locally by modulations of the alongshore winds conditioning anomalous coastal upwelling/downwelling as well as changes in the mixed-layer temperature by anomalous heat fluxes^6^. Moreover, the reduction in local wind speed will decrease the latent heat loss from the ocean to the atmosphere, thereby contributing to the development of Dakar Niños. Additional forcing may originate from the equator via the propagation of equatorial Kelvin waves (EKW) and subsequent poleward propagating coastal trapped waves (CTWs)^7^. The analysis of the satellite altimetry over the period 2002-2012 by *Illig *et al*.*^7^ revealed that some CTWs of equatorial origin propagated north up to the latitude of Liberia (~ 6°N). A downwelling (upwelling) CTW reaching the CDNI region would deepen (shoal) the thermocline and nutricline, leading to a reduction (enhancement) of local upwelling, positive (negative) SST anomalies, and ultimately anomalously low (high) NPP. Similar impacts of CTWs on SLA, SST and NPP variability have already been well demonstrated along the southwestern African coast in the Angola Benguela upwelling system^8–14^. However, the role of CTWs in the development of Dakar Niños/Niñas and their impacts on the NPP has, to our knowledge, not yet been discussed. Further, *Koseki *et al.^15^ showed, using a high resolution regional coupled model, that under a warmer climate, Dakar Niños may intensify without changing their location and timing. NPP in the CCUS can be modulated by climate modes operating in the Atlantic Ocean. The North Atlantic Oscillation (NAO)^16^, a hemispheric meridional oscillation in atmospheric mass with centers of action near Iceland and over the subtropical Atlantic^17^, has been reported to influence the upwelling intensity in all three parts of the CCUS by intensifying (reducing) the upwelling favourable winds during a positive (negative) phase^18,19^. Additionally, *Gómez-Letona *et al.^3^ showed significant correlations between the NAO index and the NPP in the seasonal upwelling area in January-February-March over the period 2003-2015. Furthermore, the Atlantic Meridional mode (AMM)^20^, characterized by an interhemispheric SST gradient in the tropical Atlantic and peaking during the upwelling season (i.e., MAM), can also impact the NPP in the CDNI region by affecting the position of the ITCZ. In fact, during a positive (negative) phase of the AMM, significantly reduced (enhanced) northeasterly trade winds generate a large-scale warming (cooling) in the tropical North Atlantic that extends to the CDNI region through a reduction (strengthening) of surface evaporative cooling^21^. Yet, additional modulation of the NPP in the CCUS may originate from other basins. For example, *Roy and Reason*^22^ reported that between 1957 and 1995, coastal SST anomalies in the southern part of the CCUS were preceded by El Niño-Southern Oscillation (ENSO) events in the Pacific by several months. The authors suggested a remote forcing through an atmospheric bridge between the Pacific and Atlantic Oceans with positive (negative) ENSO events leading to a relaxation (intensification) of the trade winds in the Atlantic affecting the coastal upwelling and highlighted by warm (cold) SST anomalies in the southern part of the CCUS. However, a previous study by *Wang *et al.^23^ showed that the relationship between ENSO and SST anomalies (SSTA) in the tropical North Atlantic (including the CDNI region) is modulated by the Atlantic multidecadal variability (AMV). Over the period 1982-2011, *Oettli *et al*.*^6^ did not find a consistent response of the SST off the coasts of Senegal and Mauritania to ENSO. Similarly, *Gómez-Letona *et al.^3^ investigated correlations between the Multivariate ENSO index and the Southern Oscillation index with the SST in the three subregions of the CCUS and did not find any significant relationships over the period 1993-2014. Yet, *López-Parages *et al.^24^ showed, using an ocean model, that over the period 1985-2009, ENSO influenced the round sardinella population biomass and distribution in the central to southern part of the CCUS. Hence, the remote effect of ENSO on the NPP in the southern part of the CCUS remains an open question.

Here, building on previous studies investigating the seasonal to decadal variability of the NPP in the CCUS^3^, we document for the first time the interannual variations in NPP occurring in the CDNI during the upwelling season (MAM) over the period 2003-2023. The link between extreme NPP events and the occurrence of Dakar Niños/Niñas is examined. Furthermore, potential remote forcing through CTW originating in the northern Gulf of Guinea or in the equatorial Atlantic, as well as the influence from different climate modes are investigated.

## Results

### Interannual NPP variability

An area of large NPP variability, defined by the standard deviation of the monthly NPP anomalies (NPPA), is observed off the coasts of Senegal and Mauritania mostly within the CDNI region (Fig. 2a). Concomitantly, this coastal region presents large variability of the SST as shown by the standard deviation of the monthly SSTA (blue contours in Fig. 2a).

The large SST variability is driven by the occurrence of extreme warm and cold events called Dakar Niños and Niñas^6^, respectively. Moreover, the NPP in the CCUS features a pronounced annual cycle with a maximum in NPP during MAM and a minimum in July-August-September (JAS; Fig. 1, Fig. 2b). However, the seasonal cycle of the standard deviation of the NPP anomalies features two peaks, a first peak in March and a second peak in May. This suggests that the variability of the NPP can be seen as a modulation of its annual cycle, i.e., an earlier or later seasonal peak in NPP and/or a smaller or larger annual maximum. The interannual variability of the NPP in MAM is preceded by large variations in SST in February-March-April (Fig. 2c). The time series of detrended anomalies of NPP averaged in the CDNI region is shown in Fig. 2d. The NPPA clearly exhibit a strong year to year variability marked by the occurrences of extreme NPP events. Since the interannual variability of the NPP is maximum in MAM, for this study, only the extreme events that peaked during MAM are considered. Therefore, nine anomalous coastal events are identified and highlighted by their corresponding years in Fig. 2d. They are classified into 3 extreme high NPP events (2012, 2015, 2016) and 6 extreme low NPP events (2005, 2008, 2010, 2020, 2021, 2023). More details on the extreme NPP events can be found in the supplementary tablesS1 and S2. Yet, to our knowledge, there are no studies in the literature that have described the interannual variability of the NPP in the CDNI region. In order to allow for significant and meaningful results we will focus on the extreme low NPP events for the rest of the study. Despite the lack of a long time series of NPP, Fig. 2d shows indications of a decadal variability signal of the NPPA in the CDNI region with prevailing phases of negative NPPA from 2003 to 2011 and after 2020 and a positive phase of NPPA between 2011 and 2020.

### Role of local processes and remotely forced CTWs

**Fig. 3:**
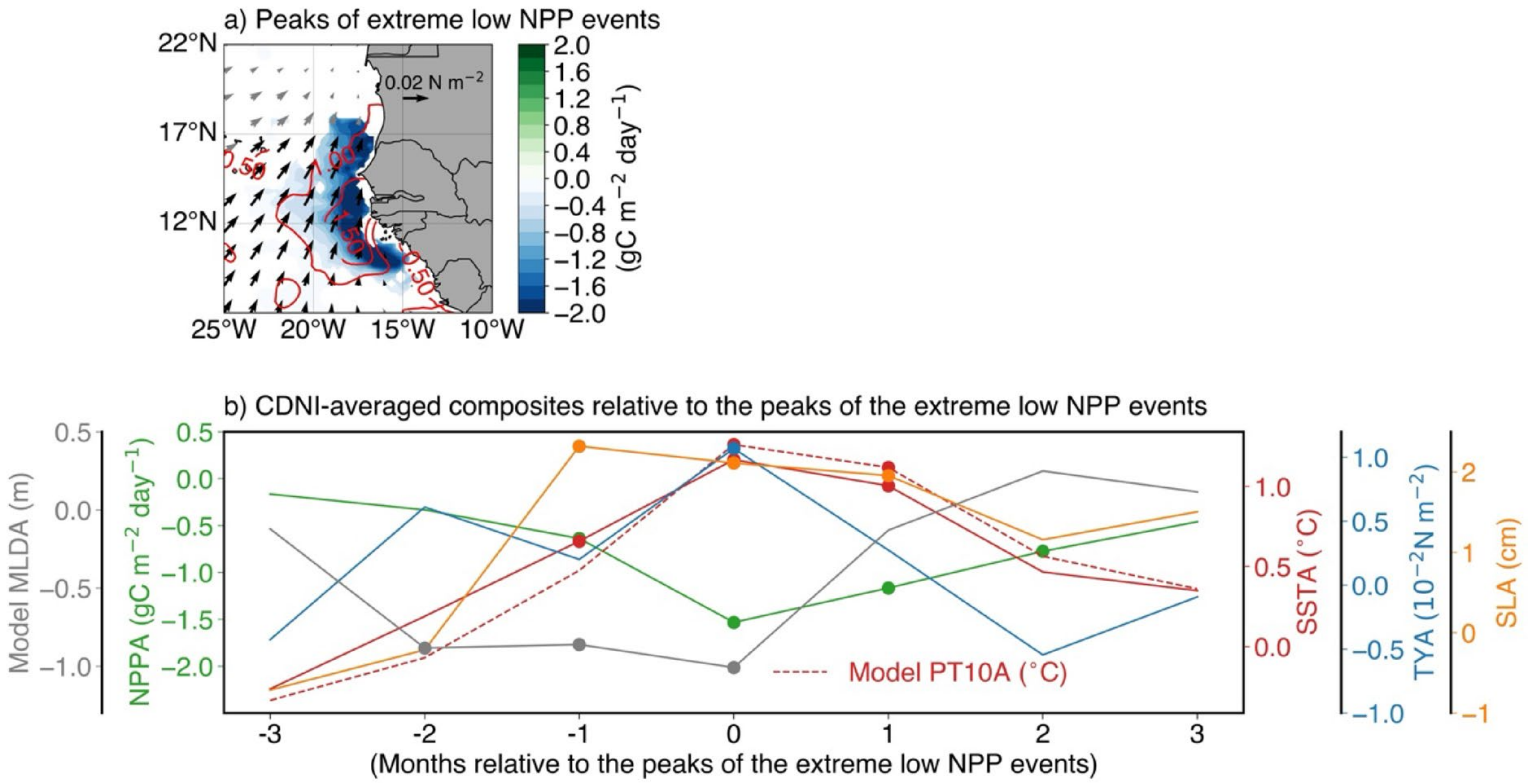
(**a**) Composite map of monthly detrended NPPA (shading), SSTA (red contours, every 0.5 °C) and ERA5 wind stress (arrows) computed from peak months of six extreme low NPP events (2005; 2008; 2010; 2020; 2021 and 2023). Shaded areas (NPPA), contours (SSTA) and black arrows (wind stress) displayed in (a) are statistically significant at 95% confidence level. (**b**) Time series of composite anomalies of the CDNI-averaged: SSTA (solid red line), NPPA (green line), meridional wind stress anomalies (TYA, blue line), sea level anomalies (SLA, orange line), model potential temperature anomalies at 10 m (PT10A, dashed red line) and model mixed layer depth (MLDA, gray line). The composite anomalies displayed in (b) are relative to the peaks of the extreme low NPP events, ranging from 3 months before to 3 months after those peaks. Values significant at the 95% confidence level are denoted by a filled circle.

To better characterize the relation between extreme low NPP events, surface wind stress and SST along the northwest African coast, Fig. 3a shows a 95% statistically significant composite analysis of detrended anomalies of those parameters relative to the peaks of the low NPP events. Details about the selected years for the composite are provided in the caption of Fig. 2.

**Fig. 4:**
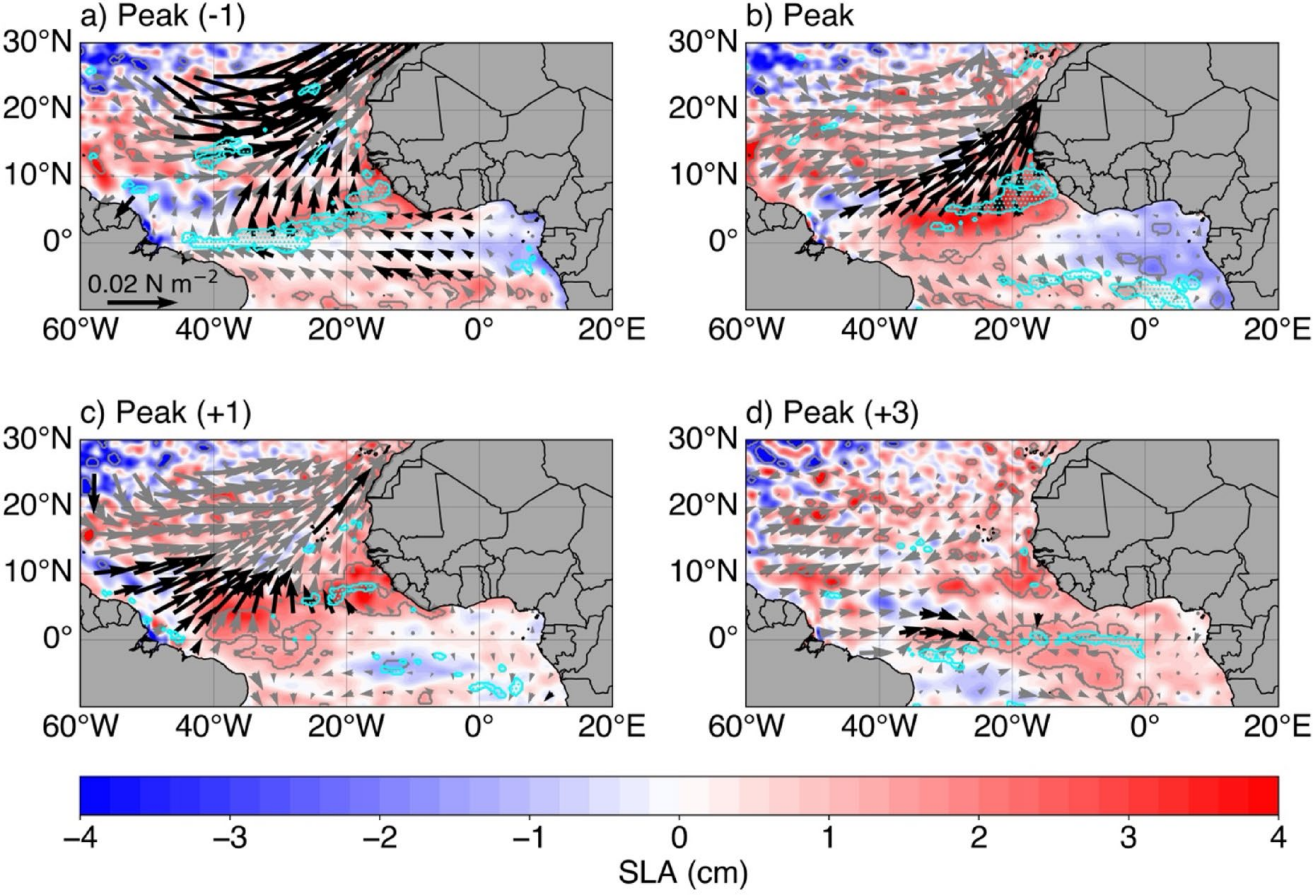
(**a**–**d**) Composite maps of monthly detrended anomalies of: SLA (shading with contours indicate areas statistically significant at 95% confidence level), negative wind stress curl (cyan contours and dots indicate areas statistically significant at 95% confidence level, see Fig. S1) and surface wind stress (arrows, with black arrows representing wind stress statistically significant at 95% confidence level). The composite anomalies are relative to the peaks of the extreme low NPP events (2005; 2008; 2010; 2020; 2021 and 2023) with in (**a**) Peak (− 1), (**b**) Peak, (**c**) Peak (+ 1) and (**d**) Peak (+ 3) representing one month before, peak, one month and 3 months after the peak months of extreme low NPP events, respectively.

The mature phase of the extreme low NPP events that peak during MAM is marked by the presence of low NPPA limited to latitudinal boundaries of the CDNI region and extending from the coast to ~ 22°W with anomalies below -2 gC m^−2^ day^−1^. The low NPPA occurs simultaneously with statistically significant warm SSTA (> 1 °C), in the CDNI region. Since the standard deviation of CDNI-averaged SSTA in FMA is around 0.71 °C, the warm SSTA that are observed during the peak of extreme low NPP events could indicate the occurrence of Dakar Niños. Therefore, Dakar Niños are linked to the reduction of NPP. Additionally, during the mature phase of the extreme low NPP events, statistically significant southwesterly anomalies over the warm SST and low NPP areas south of 17°N are observed. In fact, these reduced trade winds will induce a reduction in the offshore Ekman transport and coastal upwelling leading to a warming and negative NPPA. Averaged in the CDNI region, Fig. 3b shows the time evolution of composite anomalies of local observed SST, model potential temperature at 10 m (PT10), NPP, meridional wind stress, mixed layer depth and SLA. As expected, statistically significant positive anomalies of meridional wind stress (~ 0.01 N m^−2^) are observed only during the peak of the low NPP events. Further, positive meridional wind stress anomalies are already observed two months before the peak of low NPP events even though they are not statistically significant. This will reduce the coastal upwelling which will generate statistically significant coastal warm SSTA (statistically significant at 95% from one month before to one month after the peak). Note that additional coastal warming effect may come from reduced latent heat loss through the weaker northeasterly trade winds. Likewise, model PT10 anomalies (model PT10A) are quite consistent with observations and show significant values during the peak and one month after the peak. Simultaneously, the mixed layer is anomalously thin (less than - 0.9 m) for about 3 months (from two months prior to the peak until the peak). Note that the shoaling of the mixed layer favours a warming by the shortwave heat fluxes. In response to the reduced meridional wind stress, warm SSTA and thin mixed layer, coastal NPP is reduced with a maximum reduction of - 1.53 gC m^−2^ day^−1^ during the peak. Statistically significant low NPPA persist in the region until 2 months after the peak where they reached - 0.77 gC m^−2^ day^−1^. In addition to the low NPPA, Fig. 3b also portrays a local maximum positive anomaly of SLA (downwelling coastal SLA signal) of 2.3 cm one month before the peak of low NPP events which decays by 0.35 cm up to 2 months later. Since maximum downwelling coastal SLA signal precedes minimum negative coastal NPPA by one month, we further analyse the downwelling coastal SLA signal. Spatial composite maps of anomalies of SLA, surface wind stress and wind stress curl are shown in Fig. 4. Indeed, besides the reduced local meridional wind stress, statistically significant negative near-coastal wind stress curl anomalies (WSCA; cyan contours and dots in Fig. 4a with shading in Fig. S1a) are observed concomitantly with coastal positive anomalies of SLA one month before the peak of the low NPP events (Fig. 4a). These significant WSCA are less than - 0.15 × 10^–7^ N m^−3^ (Fig. S1a). Note that a negative near-coastal WSCA indicates a reduction of the mean near-coastal cyclonic wind stress curl, resulting in weakened Ekman suction, i.e., downwelling anomalies and reduction of the coastal upwelling. This weakened Ekman suction has contributed to coastal warming (Fig. 3b) and led to positive coastal anomalies of SLA. The statistically significant negative WSCA extend offshore to the west until the western and central equatorial Atlantic (Fig. S1a). Off equatorial negative WSCA pattern as the one observed in Fig. 4a have already been shown to be efficient to trigger downwelling Rossby waves^25^. Note that Fig. 4a also shows a C-shape like pattern about the equator in the surface wind stress anomalies which can be seen as the signature of the wind-evaporation-SST (WES) feedback^21^ indicating that the AMM is in its positive phase. Statistically significant offshore reduced northeasterly trade winds are associated with the reduction of the Azores high-pressure system (not shown, sea level pressure anomalies < -200 Pa). A strong reduction of the surface wind stress in the CDNI region is only observed during the peak of low NPP events (Fig. 4b) as shown in Fig. 3b for the meridional wind stress.

Figure 4a also portrays statistically significant easterly wind stress anomalies, spreading from ~ 5°S to the northern Gulf of Guinea (mainly coastal regions of Ghana and Côte d’Ivoire) and from 15°W to 0°N. We suggest that these easterly wind stress anomalies might provide additional forcing of downwelling coastal trapped waves (CTWs), that would propagate along the northwest African coast, deepening the thermocline and contributing to positive anomalies of SLA and SSTA in the CDNI. These downwelling CTWs also deepen the nutricline, thereby reducing the local NPP. Additionally, off the equator, part of the energy of the downwelling CTWs is transmitted westward as downwelling Rossby waves. This could explain the statistically significant positive anomalies of SLA spreading to the west at ~ 3°N.

One month later (Fig. 4b), i.e., during the peak of the low NPP events, the negative WSCA persist north of the equator (see also Fig. S1b) providing additional forcing of the downwelling Rossby wave. This downwelling Rossby wave then propagates westward and is associated with off-equatorial positive anomalies of SLA exceeding 3 cm (Figs. 4b-c). Upon reaching the South American coast, that downwelling Rossby wave will be completely reflected into an eastward propagating downwelling equatorial Kelvin wave at around two months after the peak of the low NPP events (Peak (+ 2), not shown) and will then influence the equatorial Atlantic variability one month later (Peak (+ 3), Fig. 4d).

Although not statistically significant, negative anomalies of SLA observed along the equator (east of 20°W) and along the western African coast east of 0°E mark the signature of wind-forced upwelling equatorial Kelvin waves and subsequently upwelling CTWs triggered by easterly wind stress anomalies along the equator (Fig. 4a).

## Mixed-layer heat budget analysis

**Fig. 5:**
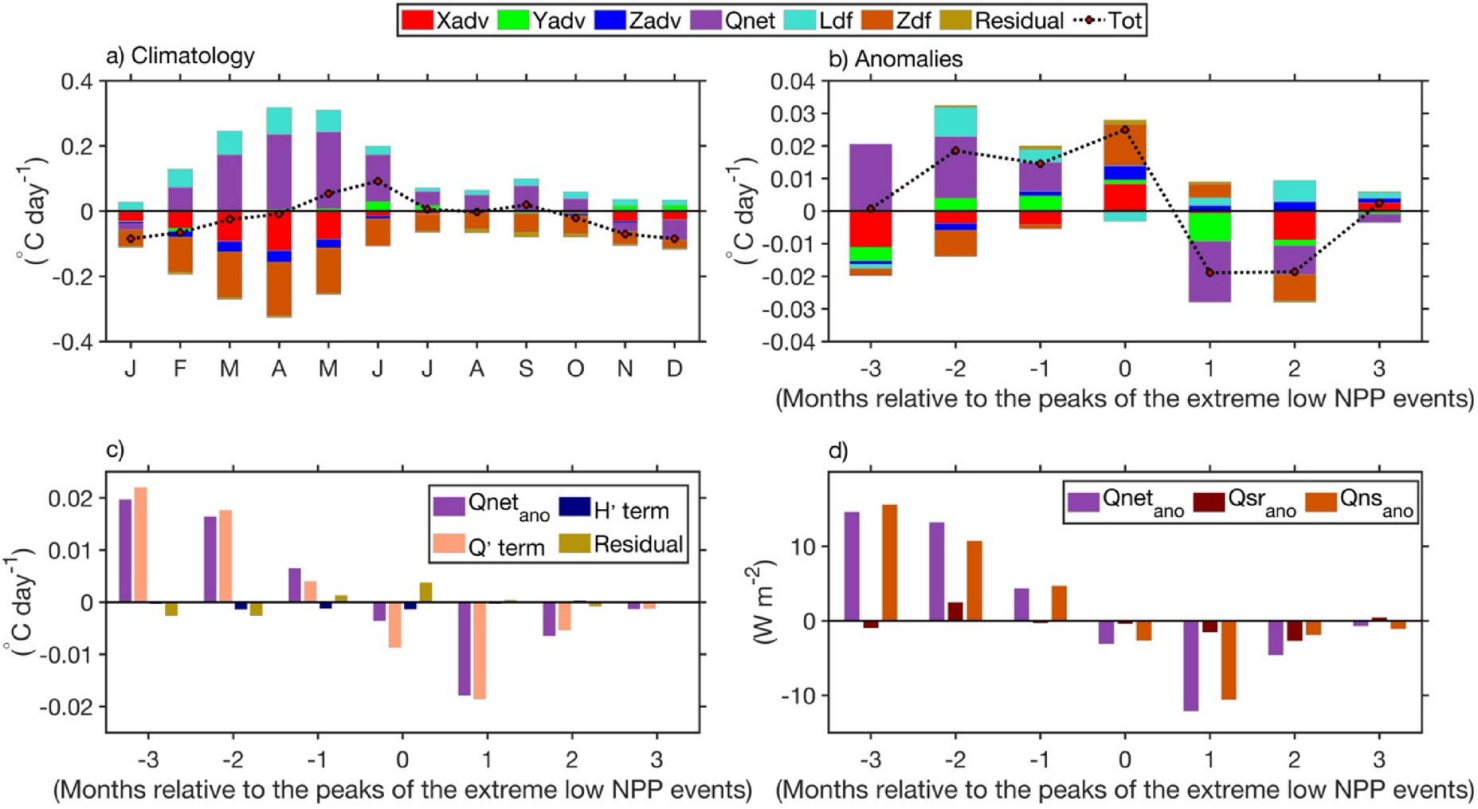
(**a**) Monthly climatology of the CDNI-averaged terms of the model mixed layer heat budget from 2003 to 2023, where Tot is the total mixed layer temperature tendency, Xadv, Yadv and Zadv are the zonal, meridional and vertical advection, respectively, and Ldf, Zdf and Qnet are the lateral and vertical diffusion and the net surface heat flux, respectively. (**b**) Time series of composite anomalies of the CDNI-averaged of each term of the model mixed layer heat budget. The composite anomalies are relative to the peak of the extreme low NPP events ranging from 3 months before to 3 months after the peak months of the extreme low NPP events. (**c**) Same as (b), but for contribution to model mixed layer temperature due to surface heat‐flux anomalies acting on climatological mixed layer depth (Q’ term), mixed layer depth anomalies acting on climatological heating/cooling (H’ term) and the residual. The detrended anomalies of the contribution to model mixed layer temperature due to net surface heat flux is also represented (Qnet_ano). (**d**) Same as (b) but for anomalies of modelled non-solar flux (Qns_ano), solar radiation flux (Qsr_ano) and net surface heat flux (Qnet_ano). Climatology and anomalies are calculated relative to the period January 2003 to December 2023.

Given that low NPP events coincide with occurrences of Dakar Niños, based on output from an ocean model (see Data and Methods section for description and validation of the model outputs), an analysis of a mixed layer heat budget is performed to investigate the drivers of mixed layer temperature under the assumption that these mechanisms may also account for the observed NPP variability. The rate of change of the mixed layer temperature anomalies is expressed in Eq. 1 and the results are shown in Fig. 5. The monthly climatology of all the terms is shown in Fig. 5a. Climatologically, the analysis shows that within the CDNI region, the net surface heat flux term is the dominant contributor to the mixed layer warming from February to October with highest contribution during MAM, the period of high NPP (Figs. 1a, 2b). Also, a minor contribution from lateral diffusion to mixed layer warming is evident throughout the year, with a maximum around MAM. In contrast, vertical diffusion, which is a proxy for vertical mixing, dominates the mixed layer cooling throughout the year, with an important contribution also during MAM. Additional cooling during MAM also arises from zonal advection, while vertical advection contributes to a lesser extent. In the CDNI region, meridional advection seems to not contribute to the mixed layer temperature during MAM, but shows minor contribution to the mixed layer warming in June, July, November and December.

Three months before the peak of the extreme low NPP events, the mixed layer is anomalously warmed (0.02 °C day^−1^, Fig. 5b) solely by the net surface heat flux, however cooled by the other terms. Note that the anomalous warming of the mixed layer is obtained by summing the anomalies from all terms with positive values. Two months prior to the peak of the extreme low NPP events, the anomalous warming of the mixed layer (0.032 °C day^−1^, Fig. 5b) is primarily driven by anomalies of the net surface heat flux explaining 58% and to a lower extent by anomalous lateral diffusion (28%), meridional advection (12%) and to an even lesser extent by anomalous residual (2%). One month later, anomalies of the net surface heat flux explaining 44% are still the main contributor to the anomalous warming of the mixed layer which has now dropped to 0.02 °C day^−1^. Note that the same contributors as for the previous month are still observed with a reduced anomalous lateral diffusion (20%), and increased anomalous meridional advection (24%) and residual (5%). However, a minor anomalous contribution from vertical advection (7%) is now observed. During the mature phase of the low NPP events, most of the anomalous warming of the mixed layer (0.028 °C day^−1^) is induced by anomalous vertical diffusion (44%) and zonal advection (30%), while anomalous meridional and vertical advection only play minor roles. This is consistent with surface warming associated with low NPP due to reduced coastal upwelling caused by significantly weakened local coastal wind stress and negative WSCA (Figs. 3, 4b, S1b). However, anomalies of the net surface heat flux largely explain the anomalous cooling of the mixed layer (damping effect) one and three months after the peak, while anomalies of the net surface heat flux, zonal advection and vertical diffusion equally contribute to the anomalous cooling of the mixed layer two months after the peak (Fig. 5b).

Since anomalies in the net surface heat flux largely contribute to the anomalous warming or cooling of the mixed layer, we have decomposed this contribution into two terms as displayed in Eq. 2 and shown in Fig. 5c. The two terms are the model’s mixed layer temperature tendency due to net surface heat flux anomalies acting on climatological mixed layer depth (Q’ term) and climatological heating/cooling applied to an anomalous mixed layer depth (H’ term) as done by *Senapati *et al.^26^. A residual which is rather small is also estimated as the difference between net surface heat fluxes and summed up contributions of the Q’ and H’ terms. Over the entire time series, results show that the Q’ term largely dominates the contribution of anomalies of the net surface heat flux to anomalous warming or cooling of the mixed layer (Fig. 5c). This indicates that anomalous warming or cooling of the mixed layer in the CDNI region during Dakar Niños or Niñas is a direct response to positive or negative anomalies of the net surface heat flux, respectively, which are largely driven by anomalies the of non-solar flux (Fig. 5d). Note that the anomalies of the non-solar flux are dominated by anomalies of the latent heat flux which are linked to surface wind speed fluctuations in the CDNI region. The anomalous solar radiation term is only playing a minor role in the CDNI region during the extreme low NPP events (Fig. 5d).

### Role of climate modes on the NPP variability in the CDNI region

Past studies have shown that in boreal spring, the northern tropical Atlantic is influenced by the large-scale climate forcing of the AMM^6^, the preceding boreal winter ENSO^22,24,27,28^, and by other modes of variability such as the NAO^28^. Hence, we have also examined whether these climate modes may also contribute to NPP variability in the CDNI region.

**Fig. 6:**
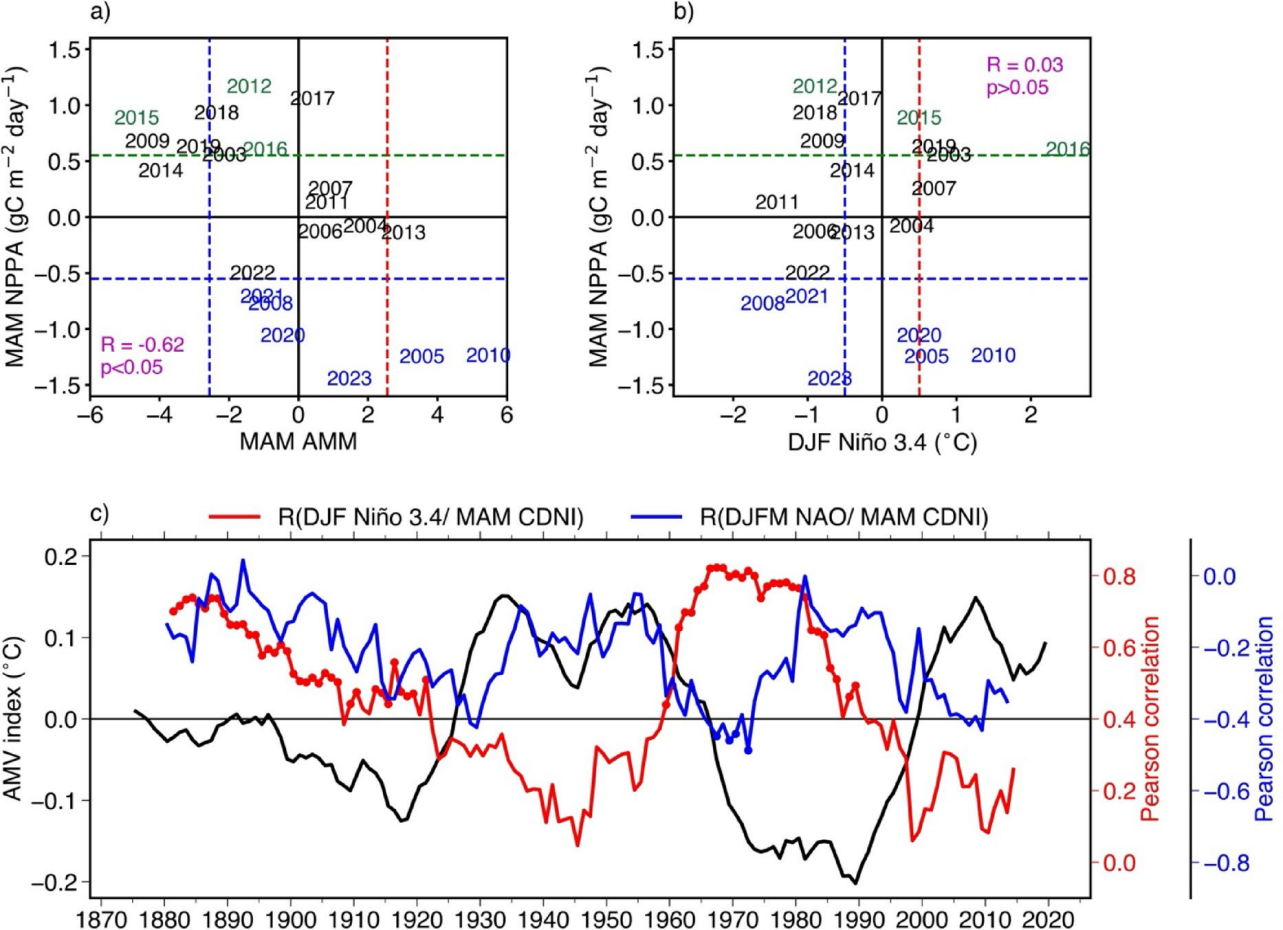
(**a**) Scatter diagram of the Atlantic Meridional Mode (AMM) index averaged during March–April-May (MAM) and CDNI-averaged NPPA during MAM. (**b**) Same as (a), but for detrended anomalies of SSTA averaged during December-January-February (DJF) in the Niño 3.4 region (120°W-170°W; 5°S-5°N) and CDNI-averaged NPPA during MAM. The AMM index is calculated following the method of *Chiang and Vimont*^20^. For ENSO intensity, the DJF values of the ONI are used. See the Data and Methods section for details on the AMM and ONI indexes. The horizontal green and blue lines in (a) and (b) represent the ± 0.8 standard deviation of the interannual CDNI-averaged NPPA (± 0.551 gC m^−2^ day^−1^). In (a) the vertical red and blue lines represent ± 1 standard deviation of the AMM index (± 2.56 °C). The vertical red and blue lines in (b) represent the threshold (± 0.5 °C) used to identify the warm and cold periods in the Niño 3.4 region. Only the extreme low and high NPP events that peak during MAM are highlighted in blue and green as in Fig. 1a. (**c**) Time series of the Atlantic multidecadal variability (AMV, black line). An 11-year centered sliding window is applied to compute annual means of the AMV. The Pearson correlation between the detrended Niño 3.4 index in DJF and the detrended CDNI in MAM (red line, with red dots indicating correlations statistically significant at 95% confidence level) is evaluated using SSTA within 21-year centrered sliding windows with the first window covering the period 1871-1891 and the last window the period 2004-2024. SSTA are defined as deviations from the monthly climatology over the period 1870–2024. The Pearson correlation between the NAO index during December-January-February-March (DJFM) and the detrended CDNI in MAM (blue line, with blue dots indicating correlations statistically significant at 95% confidence level) is evaluated using the Hurrell North Atlantic Oscillation (NAO) Index (station-based) over 21-year centered windows with the first window for 1870–1890 and the last for 2004-2024.

Figure 6a portrays the relation between the strength of the AMM events occurring during MAM and the MAM CDNI-averaged NPPA. Results show that over the period 2003-2023 and during MAM, the NPPA in the CDNI region and the AMM index are significantly (at the 95% level) linked, with a correlation of -0.62. In other words, in the CDNI region, years of positive phase of AMM are linked to anomalous surface warming in the tropical North Atlantic causing reduced northeasterly winds through the WES feedback resulting in reduced latent heat loss and coastal upwelling ultimately inducing low NPP. The opposite is observed for years of negative phase of AMM. Note that out of nine extreme NPP events recorded in the CDNI region during MAM (Fig. 2d), six extreme NPP events (66.66%, low NPP in 2005, 2010, 2023 and high NPP in 2012, 2015, 2016) have occurred during an AMM event. In addition, during MAM, SSTA in the CDNI region are linked to the AMM since the sliding correlation (with a 21-year moving window) between AMM index and CDNI-averaged detrended SSTA exhibits highly significant correlations (> 0.6), statistically significant at 95% between 1948 and 2024 (not shown). In contrast, the results from Fig. 6b clearly reveal that between 2003 and 2023, DJF ENSO events are not significantly correlated to NPP events in the CDNI region, even though some NPP events are in phase with ENSO events (for instance low (high) NPP events in 2005, 2010 (2012) in phase with El Niños (La Niña)). This means that over the period 2003-2023, other forcing mechanisms such as the local physical processes with the contribution of the remote ocean dynamics described above or the large-scale forcing of the AMM played a more important role than the remote forcing from ENSO in driving the CDNI NPP variability.

Since NPP data is only available for a short period of time and highly significant correlation (-0.68) exists between CDNI-averaged SSTA and NPPA in MAM from 2003 to 2023, we use longer time series of SSTA to investigate the link between ENSO as well as NAO with the CDNI region on longer time scales. Therefore, to further characterize the relation between DJF Niño 3.4 index and MAM SSTA, a sliding correlation (with a 21-year moving window) of detrended SSTA from the Niño 3.4 index in DJF and the CDNI in MAM is performed between 1870 and 2024 and displayed in Fig. 6c (red line). Results show that Niño 3.4 index and MAM CDNI-averaged SSTA are positively correlated over the period 1870 to 2024. However, the two indexes are significantly correlated (> 0.42, with red dots) during two periods (around 1881–1920 and late 1950s to late 1980s) and weakly correlated (not statistically significant at 95%) over the remaining periods including the period from ~ 1995 to 2014 (correlation < 0.3). The period of weak correlation (~ 1995 to 2014) between the two indexes also encompasses our study period (2003–2023) which is consistent with findings of Fig. 6b. Likewise, the same analysis is repeated using the December-January-February-March (DJFM) NAO index and MAM SSTA in the CDNI region (blue line in Fig. 6c). Results show that although the correlation between the two parameters is mostly negative all the time, it remains not significant at 95% except for the period ~ 1965 and 1975 where the correlation is below -0.4. Indeed, insignificant correlation between NAO and coastal SST was previously reported by *Cropper *et al.^18^ who showed that NAO is more strongly correlated with coastal upwelling intensity derived from winds than SST in the seasonal upwelling zone (CDNI region) between boreal winter and spring. In general, the positive (negative) phase of the NAO is associated with a strengthening (weakening) of the northeast trade winds driven by the intensification (weakening) of the Azores high-pressure system, which in turn leads to cooler (warmer) SSTA in the CDNI region. Despite this insignificant correlation, a clear multidecadal variability in the evolution of the correlation between DJFM NAO index and MAM CDNI-SSTA is observed with periods of weak and quasi-null correlation.

Since the correlations between DJF Niño 3.4/DJFM NAO indexes and MAM SSTA undergo distinct multidecadal variations (periods of high and low correlation, Fig. 6c), we have also checked if these multidecadal correlations were linked to the Atlantic Multidecadal Variability (AMV) index (black line in Fig. 6c). Interestingly, there is a clear significant correlation at 95% of - 0.60 between the time evolution of the AMV index and the sliding correlation between DJF Niño 3.4 index and MAM SSTA. This suggests that during periods of negative (positive) phases of AMV, there is a high (weak) correlation between DJF Niño 3.4 index and MAM SSTA in the CDNI region. Therefore, ENSO strongly influences the CDNI region during periods of negative AMV phases. However, the time evolution of the AMV index and the sliding correlation between DJFM NAO index and MAM SSTA shows a period in phase until about 1965 and in anti-phase thereafter.

## Summary and discussion

In this study, we have investigated the interannual variability of the NPP in the seasonal upwelling region of the CCUS, extending from 9°N to 18°N within a 2°-coastal band (named CDNI). In this region, high interannual NPP variability occurs during the main upwelling season that marks the seasonal maximum of NPP (i.e., March-April-May, Figs. 1a, 2). Over the period 2003–2023, nine extreme NPP events peaking in MAM have been identified: six extreme low events (2005, 2008, 2010, 2020, 2021, 2023) and three extreme high NPP events (2012, 2015, 2016). Given the short time series and the limited number of extreme high NPP events, this study has focused on the extreme low NPP events. Using a composite analysis of the six extreme low NPP events, the drivers of these events have been investigated.

**Fig. 7:**
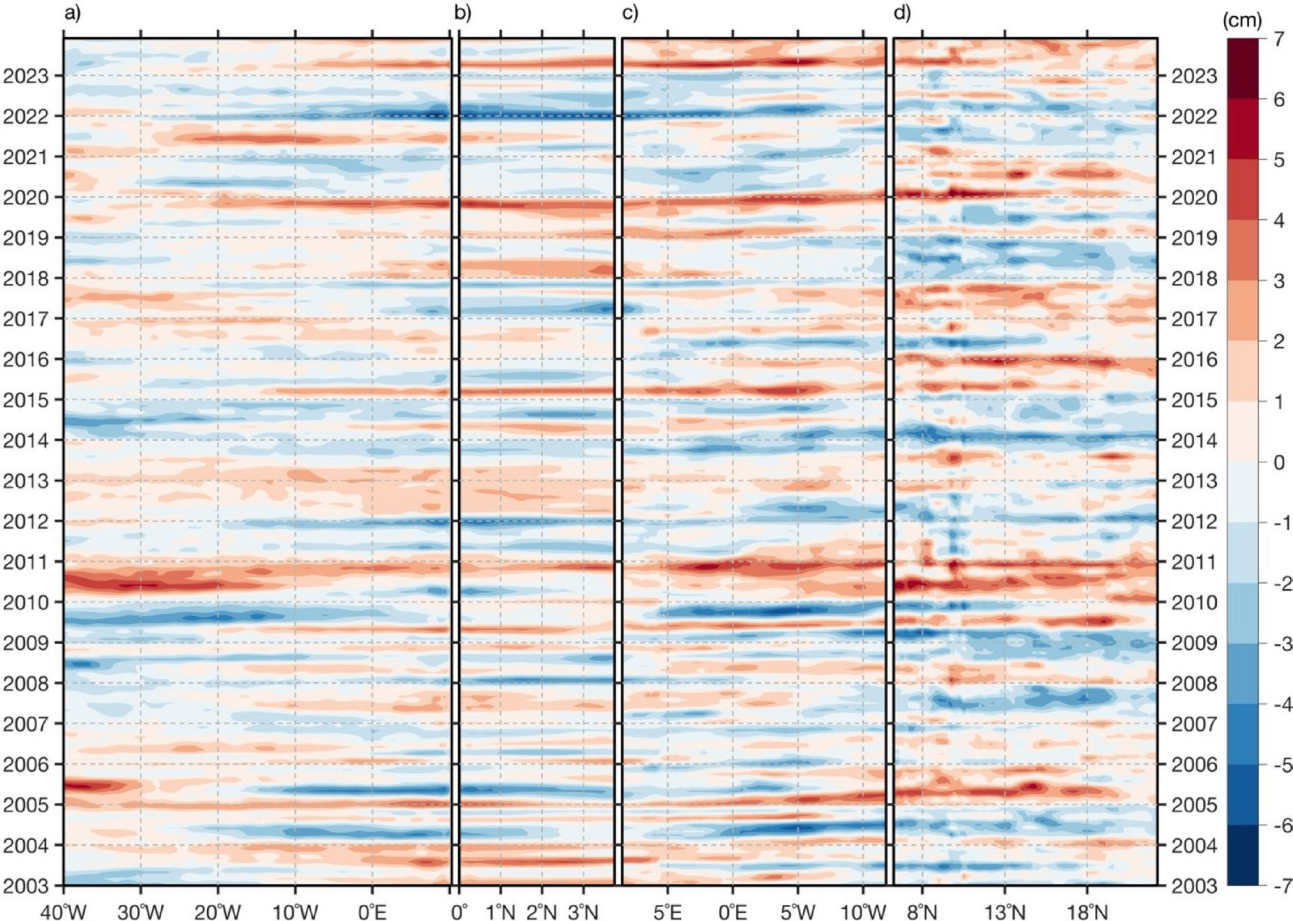
(**a**) Hovmoeller diagram of monthly detrended anomalies of SLA along the equator averaged over 1°S – 1°N. (**b**) Same as (a) with SLA averaged within 1°-coastal band from equator to around Cameroon (4°N). (**c**) Same as (a) but for SLA along the northern coast of the Gulf of Guinea averaged within 1°-coastal band along the African coast. (**d**) Same as (a) but for SLA averaged within 1°-coastal band along the northwest African coast from 7.8°N to 24°N. The anomalies are calculated relative to the monthly climatology defined over the period January 2003 to December 2023 with the decadal contribution filtered out using a high‐pass Fast Fourier Transform filter with a cut off frequency of 10 year^−1^ and the subseasonal fluctuations removed by applying a 1‐2‐1 running weighted average as in *Illig *et al*.*^7^.

Over the study period, our results have shown that extreme low NPP events are linked to both local and remote forcings. On the one hand, during the growing and peak phases of the extreme low NPP events (from two months before to the peak), the local forcing has resulted from a reduction of local wind stress and positive WSC (Ekman pumping, Figs. 4, S1). This has resulted in reduced latent heat loss and local upwelling and generated anomalously warm SSTs (Dakar Niños, *Oettli *et al*.*^6^) and positive anomalies of SLA. During these phases, anomalous shoaling of the MLD has also been observed (Fig. 3b). Our findings agree with previous studies^6,15^ which, using reanalysis and model data, found that reduced alongshore winds were among the main drivers of Dakar Niño events. On the other hand, our results suggest that easterly wind stress anomalies in the northern Gulf of Guinea (Fig. 4a) might force downwelling CTWs (highlighted by positive SLA) one month before the peak of extreme low NPP events. These downwelling CTWs would propagate along the northwest African coast and reach the CDNI, where they impact SST and NPP through deepening of the thermocline and nutricline, thus representing a remote forcing. Moreover, part of the downwelling CTW energy is transmitted westward as downwelling Rossby waves (Fig. 4). Note that the role of remotely wind-forced CTWs on the interannual SST or NPP variability in the CDNI in MAM has not been discussed in the literature before. In a previous study, *Oettli *et al*.*^6^ showed that Dakar Niños are mainly driven by local processes over the period 1982-2011, whereas *Illig *et al*.*^7^, using satellite altimetry data (2002-2012, all months), evidenced that some CTWs of equatorial origin propagate north up to the latitude of Liberia (~ 6°N) within 45 days. Similarly, *Polo *et al*.*^29^, using altimetry and model data, showed that intraseasonal (25-95 days) CTWs of equatorial origin can propagate north up to ~ 10°N. In this study, independent of the season, we show that these CTWs can propagate as far north as 24°N (for instance 2010/2011, Fig. 7), and have an impact on the coastal upwelling, thereby contributing to the development of anomalous SST events in the CDNI region. However, anomalies of SLA during extreme low NPP events in MAM show only two downwelling CTW signals of equatorial origin (2005 and 2020, see Fig. 7) with equatorial positive anomalies of SLA associated with downwelling EKW signals observed in the western or central equatorial Atlantic. Zooming into 2005 and 2020 shows that it takes ~ 5 months (from December 2004 to April 2005) and 4 months (November 2019 and February 2020) for downwelling EKWs forced in the western and central equatorial Atlantic, respectively, and subsequent downwelling CTWs to reach the CDNI region (Fig. S2). Also, note that the slopes (propagation speeds) of the positive anomalies of SLA change along the northern coast of the Gulf of Guinea (from 5°E in Figs. S2c, S2g) for both years. This change in the slope of the positive anomalies of SLA along the northern coast of the Gulf of Guinea likely reflects a shift in the dominant downwelling CTW mode from faster (low order) baroclinic modes to slower (higher order) baroclinic modes. We suggest that the slowdown of CTWs along the northern Gulf of Guinea likely reflects the influence of the narrow, steep shelf, which enhances bottom friction and favors scattering into higher baroclinic modes. Coastal stratification and river-induced density gradients (e.g., from the Niger and Volta) may provide additional contributions. The other low NPP events are linked to downwelling CTWs emanating from the northern Gulf of Guinea, except in 2021 (Fig. 7) where local positive anomalies of SLA (< 4 cm) are present between 8°N and 10°N and negative anomalies of SLA are observed along the northern coast of the Gulf of Guinea.

Additional forcing of downwelling Rossby waves (positive anomalies of SLA, Fig. 4) arises from persistent negative local WSCA (i.e., Ekman pumping, cyan contours and dots, see also Fig. S1) observed from one month before the peak to the peak of the extreme low NPP events, between ~ 5°N-10°N east of 30°W. This downwelling Rossby wave is reflected at the coast of South America as a downwelling EKW around two months after the peak of the low NPP events and takes one month to reach the eastern equatorial Atlantic (20°W-0°E) as shown in Fig. 4d, where it could be linked to a boreal summer Atlantic Niño. Similar findings in terms of location of the forcing of Rossby wave were reported by *Vallès-Casanova *et al.^25^ who investigated the influence of boreal winter Saharan dust on equatorial Atlantic variability using observational and reanalysis data. Their Fig. 6, showing the monthly lagged regression analysis of SLA and WSC onto their January-February-March dust index, revealed that negative WSCA at ~ 20°W, 3°N-6°N force a downwelling Rossby wave that will reflect into downwelling EKW in July and later precondition a winter Atlantic Niño. Note that for our study, we did not find any significant link between NPPA and Saharan dust.

Since extreme low NPP events are strongly linked to the occurrence of Dakar Niños, we have used a model mixed-layer heat budget analysis to investigate the processes driving the mixed-layer temperature during these events (Fig. 5). We found that the anomalous warming of the mixed-layer during the growing phase (three to one months before the peak) of the extreme low NPP events is predominantly explained by net surface heat flux anomalies, driven by anomalous non-solar fluxes, themselves dominated by latent heat flux anomalies which are linked to surface wind speed fluctuations. Although a shallow mixed layer (Fig. 3b) could amplify the temperature response to climatological heat fluxes (the H′ term, see Eq. 2), our analysis shows that this contribution is relatively small compared to the Q′ term (Fig. 5c). This result is in contradiction with the findings of *Oettli *et al.^6^, who found using reanalysis data, that anomalous warming of the mixed layer temperature during Dakar Niños comes from the heating of the anomalously thin surface mixed-layer even when the net surface heat flux (mostly from solar radiation) is close to climatological values. During the peak phase of the low NPP events, anomalous vertical diffusion and zonal advection are the major contributors to the anomalous warming of the mixed layer (Fig. 5b), consistent with reduced coastal upwelling caused by significantly weakened coastal wind stress and negative WSCA (Figs. 3, 4b, S1b). The demise of the extreme low NPP events is marked by anomalous cooling of the mixed layer due to anomalies of net surface heat fluxes, zonal advection and vertical diffusion.

In the CDNI region, the composite analysis shows that extreme low NPPA in MAM occur concomitantly with significant southwesterly wind anomalies and warm SSTA (Fig. 3). Past studies have suggested that the tropical North Atlantic is connected to the Equatorial Pacific via an atmospheric bridge^22,24,27,30–33^. In contrast to these previous studies, our findings show that over the entire study period, MAM NPPA were not significantly correlated with DJF Niño 3.4 index. Note that three out of nine extreme NPP events (2005, 2010, 2012) occurred in a boreal spring season following an ENSO event (Fig. 6b). Between 1870 and 2024, the 21-year sliding correlation between MAM SSTA in the CDNI region and DJF Niño 3.4 index shows multidecadal variations strongly linked to the AMV (with a significant correlation at 95% of − 0.60). During negative phases of AMV, strong and significant correlations at 95% between DJF Niño 3.4 index and MAM SSTA in the CDNI region are observed, while during positive AMV phases, as in our study period (2003–2023) and present day conditions, MAM SSTA in the CDNI region are not connected to the DJF Niño 3.4 index (Fig. 6c). These results suggest that the multidecadal correlation of Niño 3.4 with CDNI SSTA is modulated by the AMV, consistent with past studies which suggested that the AMV modulates ENSO variability: ENSO variability is intensified during negative AMV phases, while during positives phases it is weakened^34,35^. Over the study period, during MAM, in contrast to the weak link between DJF Niño 3.4 index and CDNI NPPA, coastal NPPA were strongly connected to the AMM (Fig. 6a) as also shown by *Oettli *et al.^6^ for Dakar Niños occurring in February-March-April.

A potential decadal variability of the NPP has been noticed in the CDNI region (Fig. 1d). *Gómez-Letona *et al*.*^3^ proposed that the decadal NPP variability in the seasonal upwelling zone (CDNI region) could be linked to the nutrient content of the upwelled waters, which depends on the source of the water mass: North Atlantic Central Waters (low concentration of nutrients) or South Atlantic Central Waters (high concentration of nutrients). However, longer observations of the NPP in the CCUS are needed to investigate this decadal variability.

## Data and methods

### Data

To investigate the NPP variations in the CDNI region, we use monthly mean NPP from the standard vertically generalized production model (VGPM)^36^. This product is based on Moderate Resolution Imaging Spectroradiometer (MODIS) chlorophyll, SST data and photosynthetically active radiation and estimates of the euphotic zone depth. The standard VGPM NPP data are available from July 2002 to January 2024 at a horizontal resolution of 1/6°, but has been interpolated onto a 0.25° × 0.25° spatial grid to reduce noise and match the spatial grid of other parameters such as SST. A comparison of standard VGPM to other NPP products in the CCUS is available in *Gómez-Letona *et al.^3^.

We use monthly means of SST from the Optimum interpolation SST version 2.1 (OI-SST)^37^ produced by the National Oceanic and Atmospheric Administration (NOAA) available at 0.25° horizontal resolution from September 1981 onwards. Additionally, the Hadley Centre Sea Ice and Sea Surface Temperature (HadI-SST)^38^ data set produced by the Met Office available from 1870 to present at 1° horizontal resolution is used to examine the multidecadal correlation between ENSO and the CDNI and to calculate the AMV index.

We also use the 10 m winds and wind stress from the European Centre for Medium-Range Weather Forecast (ECMWF) reanalysis version 5 (ERA5)^39^ available at 0.25° horizontal resolution from January 1940 onwards.

To investigate the multidecadal correlation between the NAO and SSTA along the coasts of Senegal and Mauritania, the Hurrell NAO index (station-based) is used^40^. This index is based on the difference of normalized sea level pressure (SLP) between Lisbon, Portugal and Stykkisholmur/Reykjavik, Iceland and is available from January 1865 to June 2023.

The *Chiang and Vimont*^20^ index for the AMM produced by the NOAA Physical Sciences Laboratory, is used to investigate its potential influence on the NPPA. The AMM index corresponds to the leading expansion coefficient of the maximum covariance analysis applied to SST between 75°W-to the West African coastline, 21°S-32°N with ENSO signal regressed out.

The AMV index is computed following a method similar to *Trenberth and Shea*^41^: monthly SSTA averaged over the global oceans (60°S-60°N) are subtracted from the monthly SSTA averaged over the North Atlantic region (80°W-0°E, 0°N-60°N).

The Ocean Niño index (ONI) produced by the Climate Prediction Centre is used to investigate the effect of ENSO in DJF on the NPPA in MAM. The ONI index is the 3-month running mean of ERSST.v5^42^ SST anomalies in the Niño 3.4 region, based on centered 30-year base periods updated every 5 years and is available from 1950 onwards.

Monthly means of SLA from the delayed-time multi-mission (all satellites merged) product are used to investigate a potential role of remote forcing onto NPPA variability in the CDNI region. SLA data are distributed by the European Union Copernicus Marine Service Information and are available at 0.25° horizontal resolution from January 1993 onwards.

### Ocean model description and validation

The mixed layer heat budget used in this study has been calculated from outputs of a regional configuration of the Nucleus for European Modeling of the Ocean program version 4.2 (NEMO-v4.2.1; *Madec *et al*.*^43^, 10.5281/zenodo.8167700). It solves the Navier‐Stokes primitive equations under spherical coordinates discretized on a horizontal Arakawa C grid and fixed vertical levels (z coordinate). This regional simulation has a 0.25° horizontal resolution and covers the tropical Atlantic (100°W–25°E, 35°S–35°N). Also, there are 75 vertical levels, with 12 levels within the first 20 m and 24 levels within the first 100 m. The momentum advection scheme is a second-order centered scheme in vector form, with a bilaplacian horizontal diffusion on momentum. Tracers are advected with a Flux Corrected Transport (FCT) scheme and an adaptative Laplacian isopycnal diffusion. The model time step is 1800s. The vertical diffusion coefficient is estimated using the generic length‐scale scheme with a k‐epsilon turbulent closure^44^. More details can be found in *Reffray *et al*.*^45^. The model is initialized in January 1958 and forced at the boundaries with interannual monthly fields from the ECMWF ORAS5 ocean reanalysis^46^. The atmospheric fluxes of momentum, heat, and freshwater at the surface are prescribed following bulk formulae^47^ using hourly fields of wind speed, atmospheric temperature, and humidity, and daily fields of longwave, shortwave radiation, and precipitation fields from the ERA5 reanalysis^39^. Daily and interannual river runoff are obtained from the ISBA-CTRIP discharge product (*Decharme *et al*.*^48^; https://zenodo.org/records/12755130). Heat budget terms (see Eq. 1) are computed online, at each model time step and vertically integrated in the mixed layer. The mixed-layer depth (MLD) is computed following a density criterion: a 0.03 kg m^−3^ difference relative to the density at 10 m^49^. Model outputs are available from 1958 to 2023. Over the period 2003-2023, monthly averages of the model PT10 have been validated against the OI-SST (Fig. S3) and all mixed layer heat budget terms analyzed (Fig. 5).

Fig. S3 shows the model PT10 validation against observations (OI-SST) from 2003 to 2023 in the northeastern tropical Atlantic and the CDNI region. Compared to observations, the model has a cold SST bias along the northwest African coast (Fig. S3a) in the CDNI region. Overall, when averaged over the CDNI region, the model exhibits a cold SST bias of - 0.47 °C. Furthermore, the model slightly overestimates the interannual SST variability near the coast in the CDNI region (Fig. S3b). The seasonality of the SST and its interannual variability in the CDNI region is overall well captured by the model (Fig S3c). The monthly climatology of the SST in the CDNI region in both model and observations features a minimum in February/March and a maximum in September/October. However, we also observe that the cold SST bias from the model is sharper during MAM compared to other seasons. In observations, the seasonal cycle of interannual SST variability in the CDNI region (Fig S3c) portrays a major (minor) peak in February (May), whereas in the model, it shows a major (minor) peak in June (February). Yet, the peak in February is slightly underestimated by the model. Quite good agreement is found between model and observational data from July till December with the interannual SST variability reaching its minimum during September/October. Model and observation time series of detrended SSTA averaged over the CDNI region exhibit a high correlation of 0.87 with root mean squared error of 0.39 °C (Fig. S3d), demonstrating especially good agreement. The model effectively accounts for the majority of the observed interannual SST fluctuations, including events such as the Dakar Niños of 2005 or 2008 (Fig. S3d). The model slightly overestimates variability in the CDNI region, with the standard deviation of interannual SSTA being 0.72 °C in observations and 0.78 °C in the model. Nevertheless, the model matches well with observations and, therefore, can be used to investigate the mixed layer heat budget in the CDNI region. Moreover, the ocean model used in this study builds on the physical configuration described in *Gévaudan*et al.^50^. Previous versions of this configuration have demonstrated skill in reproducing the interannual variability of temperature and salinity in the Tropical Atlantic^51–53^, making it a suitable tool for studying Dakar Niños.

## Methods

### Calculation of detrended anomalies and standard deviations

To obtain the monthly detrended anomalies over the period from January 2003 to December 2023, the linear trend evaluated pointwise is removed from the original data. Then the detrended data is deseasonalized by removing the climatological mean seasonal cycle evaluated over the whole period. For the NPP, after calculating the interannual NPPA between January 2003 to December 2023, for each month individually (over the entire time series), we estimate the standard deviation of the interannual NPPA which is shown in Fig. 2b, d, respectively. Similarly, in Fig. 2a, c, the standard deviation of the interannual NPPA are computed pointwise during the whole study period (only in MAM), respectively.

### Bootstrapping method

In this study, the evaluation of statistical significance of our composites was conducted employing a nonparametric bootstrap method^54,55^. Note that the bootstrap testing without replacement is chosen, which means that the months among each randomly picked group are all different. In this case, the initial monthly data set is resampled 10,000 times to form 10,000 artificial averages. These 10,000 artificial averages are then sorted in ascending order leading to a distribution. Therefore, each grid point (Figs. 3a, 4) or each value of the time series (Fig. 3b) of the composite will be statistically significant at 95% if its value fell outside the 2.5th–97.5th percentile range of this distribution (e.g. thick gray line in Fig. 4). A similar method was used in *Imbol Koungue *et al.^53^, but for statistical significance at 90%.

### Pearson correlation

Pearson correlation coefficients in Fig. 6c were computed to quantify linear relationships among the variables. Statistical significance at the 95% confidence level was assessed using a two-tailed Student’s t-test. Because the correlation coefficients were calculated using 21-year centred sliding windows (yielding 20 degrees of freedom), correlation coefficients exceeding ± 0.42 (the critical threshold) are therefore considered statistically significant.

### Model mixed layer heat balance

To understand the physical processes that drive the mixed layer temperature fluctuations during the extreme low NPP events, a mixed layer heat budget computed online is analyzed. The mixed layer heat budget is expressed as follows^56,57^:

1 $$\begin{gathered} < \partial_{t} T > = - < \, u\partial_{x} T \, > - < \, v\partial_{y} T \, > - < \, w\partial_{z} T \, > + < \, Ldiff\left( T \right) \, > \hfill \\ + < \frac{{Q_{ns} + Q_{sr} \left( {1 - f_{z = - H} } \right)}}{{\rho_{0} c_{p} H}} > - \frac{1}{H}(Kz_{{}} \partial zT)z = - h \hfill \\ \end{gathered}$$

with < $$\cdot$$ >  = $$\frac{1}{H}\mathop \smallint \limits_{ - H}^{0} .\partial z$$.

where *T* represents the model potential temperature, (*u, v, w*) are the velocity components, *Ldiff(T)* represents the lateral diffusion operator (Ldf), $\frac{1}{H}$*(K*_*z*_*∂*_*z*_*T)* is the vertical mixing (Zdf) with *K*_*z*_ the vertical diffusion coefficient for tracers. *H* is the MLD, $\rho_{0}$ is the seawater density (i.e., 1027 kg/m^3^) and $c_{p}$ the specific heat capacity of water (i.e., 4000 J/(kg K)). Here, $Q_{ns}$ and *Q*_*sr*_ are respectively the non-solar (latent, sensible and longwave heat fluxes) and solar components of the air–sea heat flux (shortwave radiation), and *f*_*z* = *-H*_ is the fraction of the shortwave radiation that reaches the MLD. *Tot (*< *∂*_*t*_*T* >*)* represents the total mixed layer temperature tendency and *Qnet (*$\frac{{Q_{ns} + Q_{sr} \left( {1 - f_{z = - H} } \right)}}{{\rho_{0} c_{p} H}}$*)* is the air–sea heat flux storage in the mixed layer, Xadv (< *u∂*_*x*_*T* >), Yadv (< *v∂*_*y*_*T* >), Zadv (< *w∂*_*z*_*T* >) are the zonal, meridional and vertical advections, respectively.

To further explore the heat flux contributions to the mixed layer temperature anomalies, we decompose the contribution of the air–sea heat flux storage in the mixed layer (see Eq. 1) into two parts (as done in *Senapati *et al*.*^26^) as follows:

2 $$\delta \left( {\frac{{ Q_{ns} + Q_{sr} \left( {1 - f_{z = - h} } \right)}}{{\rho_{0} c_{p} H}}} \right) = \delta \left( {\frac{Q}{{\rho_{0} c_{p} H}}} \right) = \frac{\delta Q}{{\rho_{0} c_{p} \overline{H}}} - \frac{{\delta H\overline{Q}}}{{\rho_{0} c_{p} \overline{H}^{2} }} + {\mathrm{Residual}}$$

with $\delta ()$ and the overbar representing anomalies and climatology, respectively. The term on the left-hand side represents the detrended anomalies of the air–sea heat flux storage in the mixed layer. The first term on the right-hand side indicates the contribution to the model mixed layer temperature anomalies due to net surface heat flux anomalies acting on climatological mixed layer depth (Q’ term = $\frac{\delta Q}{{\rho_{0} c_{p} \overline{H}}}$). The second term on the right-hand side represents the contribution to the model mixed layer temperature anomalies due to climatological heating/cooling applied to an anomalous mixed layer depth (H’ term = $\frac{{\delta H\overline{Q}}}{{\rho_{0} c_{p} \overline{H}^{2} }}$).

## Supplementary Information

Below is the link to the electronic supplementary material.

Supplementary Material 1

## Data Availability

In the following we provide links to the different datasets used for the study: OI-SST : https://psl.noaa.gov/data/gridded/data.noaa.oisst.v2.highres.html; ERA5: https://cds.climate.copernicus.eu/datasets/reanalysis-era5-single-levels-monthly-means?tab=download HadI-SST: https://www.metoffice.gov.uk/hadobs/hadisst/ Standard VGPM NPP: https://orca.science.oregonstate.edu/1080.by.2160.monthly.hdf.vgpm.m.chl.m.sst.php Hurrell NAO station-based index: https://climatedataguide.ucar.edu/climate-data/hurrell-north-atlantic-oscillation-nao-index-station-based AMM index: https://psl.noaa.gov/data/timeseries/month/AMM/ ONI ENSO index: https://www.cpc.ncep.noaa.gov/products/analysis_monitoring/ensostuff/ONI_v5.php SLA: https://marine.copernicus.eu/

## References

[1] Pauly, D. & Christensen, V. Primary production required to sustain global fisheries. *Nature* **374**, 255–257. 10.1038/374255a0 (1995).

[2] Failler, P. Climate variability and food security in Africa: The case of small s in West Africa. *J. Fish. Livest. Prod.*10.4172/2332-2608.1000122 (2014).

[3] Gómez-Letona, M., Ramos, A. G., Coca, J. & Arístegui, J. Trends in primary production in the canary current upwelling system—A regional perspective comparing remote sensing models. *Front. Mar. Sci.*10.3389/fmars.2017.00370 (2017).

[4] Davis, R. E., Hayden, B. P., Gay, D. A., Phillips, W. L. & Jones, G. V. The North Atlantic subtropical anticyclone. *J. Clim.* **10**, 728–744. 10.1175/1520-0442(1997)010%3c0728:Tnasa%3e2.0.Co;2 (1997).

[5] Sylla, A., Mignot, J., Capet, X. & Gaye, A. T. Weakening of the Senegalo-Mauritanian upwelling system under climate change. *Clim. Dyn.* **53**, 4447–4473. 10.1007/s00382-019-04797-y (2019).

[6] Oettli, P., Morioka, Y. & Yamagata, T. A regional climate mode discovered in the North Atlantic: Dakar Nino/Nina. *Sci. Rep.* **6**, 18782. 10.1038/srep18782 (2016). 26739121 10.1038/srep18782 PMC4704055

[7] Illig, S., Djakouré, S. & Mitchodigni, T. Influence of the remote equatorial dynamics on the interannual variability along the northern coast of the Gulf of Guinea. *J. Geophys. Res. Oceans*10.1029/2024jc021011 (2024).

[8] Bachèlery, M. L., Illig, S. & Dadou, I. Forcings of nutrient, oxygen, and primary production interannual variability in the southeast Atlantic ocean. *Geophys. Res. Lett.* **43**, 8617–8625. 10.1002/2016gl070288 (2016).

[9] Bachèlery, M.-L., Illig, S. & Rouault, M. Interannual coastal trapped waves in the Angola-Benguela upwelling system and Benguela Niño and Niña events. *J. Mar. Syst.*10.1016/j.jmarsys.2019.103262 (2020).

[10] Imbol Koungue, R. A., Illig, S. & Rouault, M. Role of interannual Kelvin wave propagations in the equatorial Atlantic on the Angola Benguela Current system. *J. Geophys. Res. Oceans* **122**, 4685–4703. 10.1002/2016jc012463 (2017).

[11] Imbol Koungue, R. A. et al. Drivers and impact of the 2021 extreme warm event in the tropical Angolan upwelling system. *Sci Rep* **14**, 16824. 10.1038/s41598-024-67569-7 (2024). 39039265 10.1038/s41598-024-67569-7 PMC11263681

[12] Imbol Koungue, R. A. & Brandt, P. Impact of intraseasonal waves on Angolan warm and cold events. *J. Geophys. Res. Oceans* **126**, e2020JC017088. 10.1029/2020jc017088 (2021).

[13] Illig, S. & Bachelery, M. L. Propagation of subseasonal equatorially-forced coastal trapped waves down to the Benguela upwelling system. *Sci. Rep.* **9**, 5306. 10.1038/s41598-019-41847-1 (2019). 30923330 10.1038/s41598-019-41847-1 PMC6438976

[14] Körner, M. et al. Coastal trapped waves and tidal mixing control primary production in the tropical Angolan upwelling system. *Sci. Adv.* **10**, 6686. 10.1126/sciadv.adj6686 (2024). 10.1126/sciadv.adj6686 PMC10816703 38277464

[15] Koseki, S. et al. Dakar Niño under global warming investigated by a high-resolution regionally coupled model. *Earth Syst. Dyn.* **15**, 1401–1416. 10.5194/esd-15-1401-2024 (2024).

[16] Walker, G. & Bliss, E. Memoirs of the royal meteorological society. *World Weather V* **4**, 53–84 (1932).

[17] Visbeck, M. H., Hurrell, J. W., Polvani, L. & Cullen, H. M. The North Atlantic Oscillation: Past, present, and future. *Proc. Natl. Acad. Sci.* **98**, 12876–12877. 10.1073/pnas.231391598 (2001). 11687629 10.1073/pnas.231391598 PMC60791

[18] Cropper, T. E., Hanna, E. & Bigg, G. R. Spatial and temporal seasonal trends in coastal upwelling off Northwest Africa, 1981–2012. *Deep Sea Res. Part I* **86**, 94–111. 10.1016/j.dsr.2014.01.007 (2014).

[19] Benazzouz, A. et al. An improved coastal upwelling index from sea surface temperature using satellite-based approach—The case of the canary current upwelling system. *Cont. Shelf Res.* **81**, 38–54. 10.1016/j.csr.2014.03.012 (2014).

[20] Chiang, J. C. H. & Vimont, D. J. Analogous Pacific and Atlantic meridional modes of tropical atmosphere-ocean variability. *J. Clim.* **17**, 4143–4158. 10.1175/JCLI4953.1 (2004).

[21] Amaya, D. J., DeFlorio, M. J., Miller, A. J. & Xie, S.-P. WES feedback and the Atlantic meridional mode: Observations and CMIP5 comparisons. *Clim. Dyn.* **49**, 1665–1679. 10.1007/s00382-016-3411-1 (2016).

[22] Roy, C. & Reason, C. ENSO related modulation of coastal upwelling in the eastern Atlantic. *Prog. Oceanogr.* **49**, 245–255. 10.1016/S0079-6611(01)00025-8 (2001).

[23] Wang, L., Yu, J.-Y. & Paek, H. Enhanced biennial variability in the Pacific due to Atlantic capacitor effect. *Nat. Commun.* **8**, 14887. 10.1038/ncomms14887 (2017). 28317857 10.1038/ncomms14887 PMC5364382

[24] López-Parages, J. et al. El Niño as a predictor of round sardinella distribution along the northwest African coast. *Prog. Oceanogr.*10.1016/j.pocean.2020.102341 (2020).

[25] Vallès-Casanova, I., Adam, O. & Martín Rey, M. Influence of winter Saharan dust on equatorial Atlantic variability. *Commun. Earth Environ.*10.1038/s43247-024-01926-2 (2025).

[26] Senapati, B., O’Reilly, C. H. & Robson, J. Pivotal role of mixed-layer depth in tropical Atlantic multidecadal variability. *Geophys. Res. Lett.*10.1029/2024gl110057 (2024).

[27] Saravanan, R. & Chang, P. Interaction between tropical Atlantic variability and El Niño-Southern oscillation. *J. Clim.* **13**, 2177–2194. 10.1175/1520-0442(2000)013%3c2177:IBTAVA%3e2.0.CO;2 (2000).

[28] Häkkinen, S. & Mo, K. C. The low-frequency variability of the tropical Atlantic ocean. *J. Clim.* **15**, 237–250. 10.1175/1520-0442(2002)015%3c0237:TLFVOT%3e2.0.CO;2 (2002).

[29] Polo, I., Lazar, A., Rodriguez-Fonseca, B. & Arnault, S. Oceanic Kelvin waves and tropical Atlantic intraseasonal variability: 1. Kelvin wave characterization. *J. Geophys. Res. Oceans*10.1029/2007jc004495 (2008).

[30] Alexander, M. A. et al. The atmospheric bridge: The influence of ENSO teleconnections on air-sea interaction over the global oceans. *J. Clim.* **15**, 2205–2231. 10.1175/1520-0442(2002)015%3c2205:TABTIO%3e2.0.CO;2 (2002).

[31] Chang, P., Saravanan, R. & Ji, L. Tropical Atlantic seasonal predictability: The roles of El Niño remote influence and thermodynamic air-sea feedback. *Geophys. Res. Lett.*10.1029/2002gl016119 (2003).

[32] Mo, K. C. & Häkkinen, S. Interannual variability in the tropical Atlantic and linkages to the Pacific. *J. Clim.* **14**, 2740–2762. 10.1175/1520-0442(2001)014%3c2740:IVITTA%3e2.0.CO;2 (2001).

[33] Huang, B. Remotely forced variability in the tropical Atlantic Ocean. *Clim. Dyn.* **23**, 133–152. 10.1007/s00382-004-0443-8 (2004).

[34] Dong, B., Sutton, R. T. & Scaife, A. A. Multidecadal modulation of El Niño-Southern Oscillation (ENSO) variance by Atlantic Ocean sea surface temperatures. *Geophys. Res. Lett.*10.1029/2006gl025766 (2006).

[35] No, H.-H., Kang, I.-S. & Kucharski, F. ENSO amplitude modulation associated with the mean SST changes in the tropical central Pacific induced by Atlantic multidecadal oscillation. *J. Clim.* **27**, 7911–7920. 10.1175/jcli-d-14-00018.1 (2014).

[36] Behrenfeld, M. J. & Falkowski, P. G. Photosynthetic rates derived from satellite-based chlorophyll concentration. *Limnol. Oceanogr* **42**, 1–20. 10.4319/lo.1997.42.1.0001 (1997).

[37] Reynolds, R. W. et al. Daily high-resolution-blended analyses for sea surface temperature. *J. Clim.* **20**, 5473–5496. 10.1175/2007JCLI1824.1 (2007).

[38] Rayner, N. A. et al. Global analyses of sea surface temperature, sea ice, and night marine air temperature since the late nineteenth century. *J. Geophys. Res. Atmos.* **108**, 4407. 10.1029/2002JD002670 (2003).

[39] Hersbach, H. et al. The ERA5 global reanalysis. *Q. J. R. Meteorol. Soc.* **146**, 1999–2049. 10.1002/qj.3803 (2020).

[40] Hurrell, J. W. Decadal trends in the north Atlantic oscillation: Regional temperatures and precipitation. *Science* **269**, 676–679. 10.1126/science.269.5224.676 (1995). 17758812 10.1126/science.269.5224.676

[41] Trenberth, K. E. & Shea, D. J. Atlantic hurricanes and natural variability in 2005. *Geophys. Res. Lett.*10.1029/2006gl026894 (2006).

[42] Zhang, H.-M. et al. Extended reconstructed sea surface temperature, version 5 (ERSSTv5): Upgrades, validations, and intercomparisons. *J. Clim.* **30**, 8179–8205. 10.1175/jcli-d-16-0836.1 (2017).

[43] Gurvan, M. *et al.* (Zenodo, 2023).

[44] Umlauf, L. & Burchard, H. A generic length-scale equation for geophysical turbulence models. *J. Mar. Res.* **61**, 235–265. 10.1357/002224003322005087 (2003).

[45] Reffray, G., Bourdalle-Badie, R. & Calone, C. Modelling turbulent vertical mixing sensitivity using a 1-D version of NEMO. *Geosci. Model Dev.* **8**, 69–86. 10.5194/gmd-8-69-2015 (2015).

[46] Zuo, H., Balmaseda, M. A., Tietsche, S., Mogensen, K. & Mayer, M. The ECMWF operational ensemble reanalysis–analysis system for ocean and sea ice: a description of the system and assessment. *Ocean Sci.* **15**, 779–808. 10.5194/os-15-779-2019 (2019).

[47] Large, W. G. & Yeager, S. G. The global climatology of an interannually varying air–sea flux data set. *Clim. Dyn.* **33**, 341–364. 10.1007/s00382-008-0441-3 (2008).

[48] Decharme, B. et al. Recent changes in the ISBA-CTRIP land surface system for use in the CNRM-CM6 climate model and in global off-line hydrological applications. *J. Adv. Model. Earth Syst.* **11**, 1207–1252. 10.1029/2018MS001545 (2019).

[49] de Boyer Montégut, C., Madec, G., Fischer, A. S., Lazar, A. & Iudicone, D. Mixed layer depth over the global ocean: An examination of profile data and a profile-based climatology. *J. Geophys. Res. Oceans* **109**, C12003. 10.1029/2004JC002378 (2004).

[50] Gévaudan, M., Jouanno, J., Aumont, O. & Boutin, J. On the importance of riverine organic matter for the amazon plume: A modeling study. *J. Geophys. Res. Oceans* **130**, e2024JC021527. 10.1029/2024JC021527 (2025).

[51] Awo, F. M. et al. Sea surface salinity signature of the tropical Atlantic interannual climatic modes. *J. Geophys. Res. Oceans* **123**, 7420–7437. 10.1029/2018jc013837 (2018).

[52] Gévaudan, M., Durand, F. & Jouanno, J. Influence of the amazon-orinoco discharge interannual variability on the western tropical Atlantic salinity and temperature. *J. Geophys. Res. Oceans* **127**, e2022JC018495. 10.1029/2022JC018495 (2022).

[53] Imbol Koungue, R. A., Rouault, M., Illig, S., Brandt, P. & Jouanno, J. Benguela Niños and Benguela Niñas in forced ocean simulation from 1958 to 2015. *J. Geophys. Res. Oceans* **124**, 5923–5951. 10.1029/2019jc015013 (2019).

[54] McClave, J. T. & Dietrich, F. H. *Statistics* (Dellen Publishing Company, 1991).

[55] Efron, B. & Tibshirani, R. J. *An Introduction to the Bootstrap* (Taylor & Francis, 1994).

[56] Jouanno, J., Marin, F., du Penhoat, Y., Sheinbaum, J. & Molines, J.-M. Seasonal heat balance in the upper 100 m of the equatorial Atlantic Ocean. *J. Geophys. Res.*10.1029/2010jc006912 (2011).

[57] Jouanno, J., Hernandez, O. & Sanchez-Gomez, E. Equatorial Atlantic interannual variability and its relation to dynamic and thermodynamic processes. *Earth Syst Dyn.* **8**, 1061–1069. 10.5194/esd-8-1061-2017 (2017).

