# Supplementary material for: Interannual variability of net primary productivity in the northwest African coastal upwelling system and their relation to Dakar Niños
Source: Sci Rep. 2025 Dec 16;15:43875. doi: 10.1038/s41598-025-31860-y (PMC12708759; doi:10.1038/s41598-025-31860-y)
Supplement: Supplementary file 1 — Supplementary Material 1 [file 41598_2025_31860_MOESM1_ESM.docx]

**Supplementary figures**


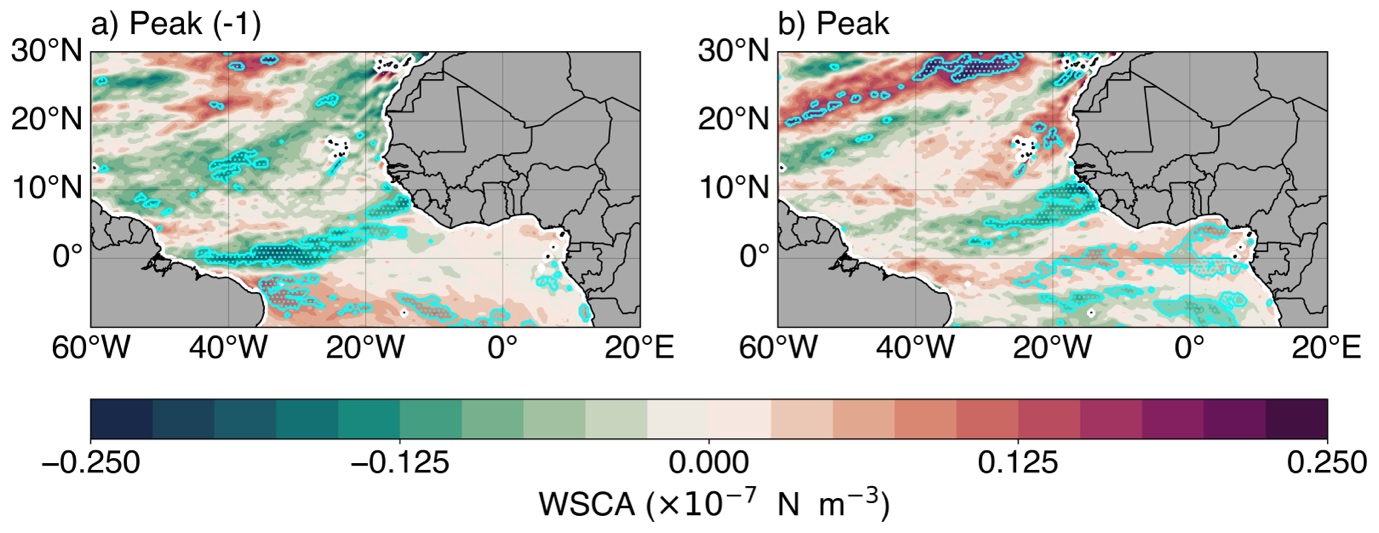


Figure S1: (a-b) Composite maps of monthly detrended wind stress curl anomalies (WSCA, shading) computed from the six extreme low NPP events (2005; 2008; 2010; 2020; 2021 and 2023). The cyan contours and dots indicate areas statistically significant at 95% confidence level. The composite anomalies are relative to the peak months of the extreme low NPP events with in (a) Peak (-1) and (b) Peak, representing one month before the peak months of extreme low NPP events, and the peak months of extreme low NPP events, respectively.


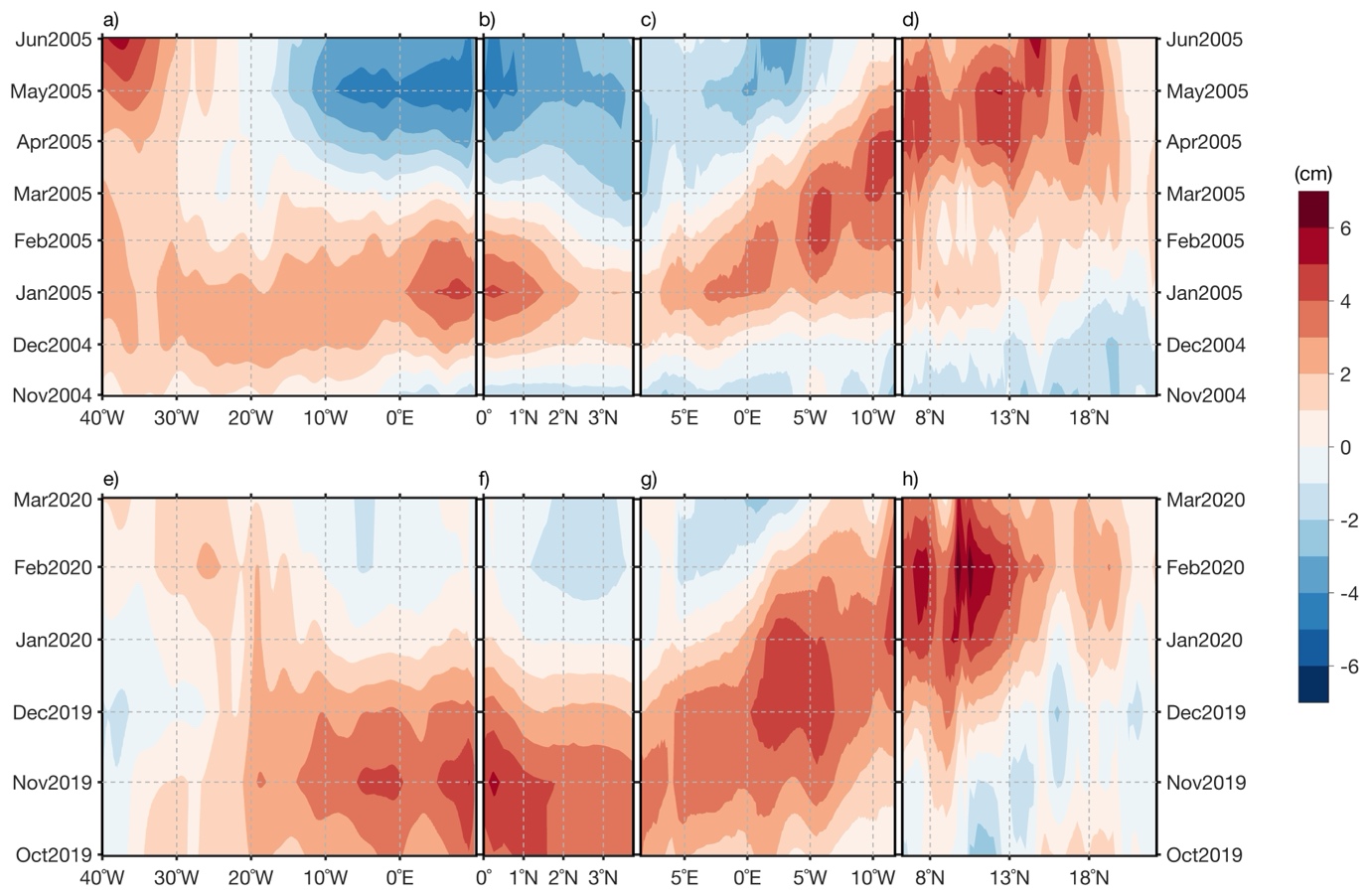


Figure S2: (a-d) Same as Fig. 7, but zoom in from November 2004 to June 2005. (e-h) Same as (a-d), but zoom in from October 2019 to March 2020.


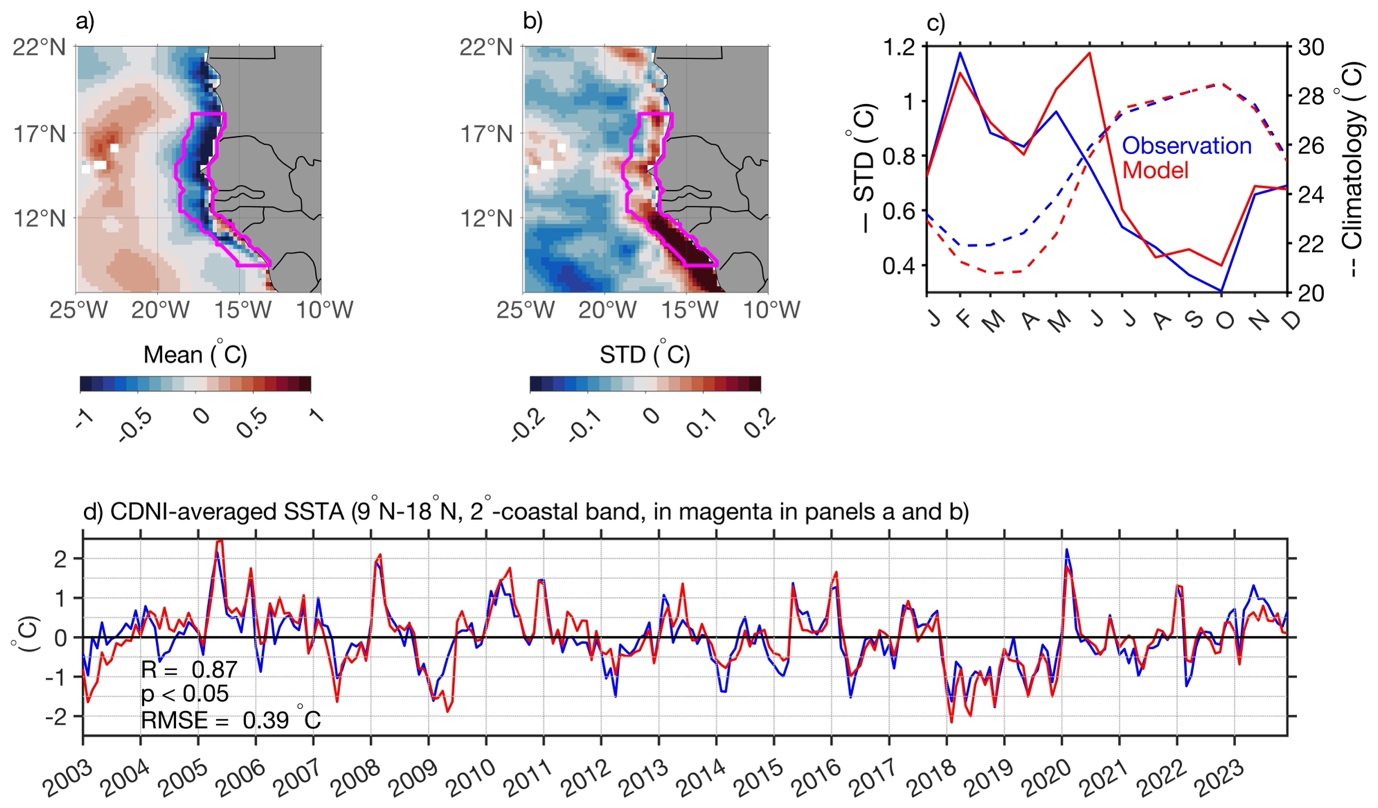


Figure S3: Model validation for SSTA in the CDNI region from 2003 to 2023. (a) Mean monthly model PT10 minus mean OI-SST. (b) Standard deviation of detrended model PT10A minus the standard deviation of detrended anomalies of OI-SST. The magenta region in (a) and (b) represents the CDNI region (9°N-18°N, 2°-coastal band). (c) Seasonal cycle of the standard deviation of CDNI-averaged detrended model PT10A (red) and OI-SSTA (blue); and climatology of CDNI-averaged model PT10 (red) and CDNI-averaged OI-SST (blue). (d) Monthly detrended anomalies of CDNI-averaged model PT10 (red) and CDNI-averaged OI-SST (blue).

**Supplementary Tables**

| **Events** | **Start date**  (YYYY-MM) | **End date**  (YYYY-MM) | **Overall peak date**  (YYYY-MM) | **Overall peak value (gC m-2 day-1)** | **Peak Date in MAM**  (YYYY-MM) | **Peak Value in MAM (gC m-2 day-1)** |
| --- | --- | --- | --- | --- | --- | --- |
| **2005** | **2005-03** | **2005-06** | **2005-05** | **-1.70** | **2005-05** | **-1.70** |
| **2008** | **2008-02** | **2008-03** | **2008-03** | **-1.41** | **2008-03** | **-1.41** |
| 2008 | 2008-05 | 2008-06 | 2008-06 | -0.97 | 2008-05 | -0.56 |
| 2009 | 2009-12 | 2010-01 | 2010-01 | -0.72 | None | None |
| **2010** | **2010-03** | **2010-06** | **2010-03** | **-1.48** | **2010-03** | **-1.48** |
| 2010 | 2010-12 | 2011-01 | 2011-01 | -0.71 | None | None |
| 2016 | 2016-01 | 2016-02 | 2016-01 | -0.94 | None | None |
| **2020** | **2020-01** | **2020-04** | **2020-02** | **-1.91** | **2020-03** | **-1.90** |
| **2021** | **2021-03** | **2021-04** | **2021-03** | **-1.20** | **2021-03** | **-1.20** |
| 2022 | 2022-12 | 2023-01 | 2023-01 | -0.67 | None | None |
| **2023** | **2023-03** | **2023-06** | **2023-03** | **-1.49** | **2023-03** | **-1.49** |

Table S1: Details of all the CDNI extreme coastal low NPP events showing the event periods, peak months and values of the event, start date and end date.

| **Events** | **Start date**  (YYYY-MM) | **End date**  (YYYY-MM) | **Overall peak date**  (YYYY-MM) | **Overall peak value (gC m-2 day-1)** | **Peak Date in MAM**  (YYYY-MM) | **Peak Value in MAM (gC m-2 day-1)** |
| --- | --- | --- | --- | --- | --- | --- |
| 2009 | 2009-02 | 2009-03 | 2009-02 | 0.75 | 2009-03 | 0.63 |
| **2011** | **2011-11** | **2012-04** | **2012-03** | **2.47** | **2012-03** | **2.47** |
| **2015** | **2015-01** | **2015-04** | **2015-04** | **2.63** | **2015-04** | **2.63** |
| **2016** | **2016-05** | **2016-06** | **2016-05** | **2.23** | **2016-05** | **2.23** |
| 2017 | 2017-02 | 2017-05 | 2017-02 | 1.31 | 2017-03 | 1.14 |
| 2017 | 2017-12 | 2018-01 | 2018-01 | 0.96 | None | None |
| 2018 | 2018-05 | 2018-06 | 2018-06 | 1.37 | 2018-05 | 1.18 |
| 2018 | 2018-11 | 2019-02 | 2019-02 | 0.82 | None | None |
| 2019 | 2019-05 | 2019-07 | 2019-06 | 2.09 | 2019-05 | 1.25 |

Table S2: Same as Table S1, but for all the CDNI extreme coastal high NPP events.
